# The deubiquitinase OTUB1 augments NF-κB-dependent immune responses in dendritic cells in infection and inflammation by stabilizing UBC13

**DOI:** 10.1038/s41423-020-0362-6

**Published:** 2020-02-05

**Authors:** Floriana Mulas, Xu Wang, Shanshan Song, Gopala Nishanth, Wenjing Yi, Anna Brunn, Pia-Katharina Larsen, Berend Isermann, Ulrich Kalinke, Antonio Barragan, Michael Naumann, Martina Deckert, Dirk Schlüter

**Affiliations:** 1 Institute of Medical Microbiology and Hospital Hygiene, Otto-von-Guericke University Magdeburg, 39120 Magdeburg, Germany; 2 Institute of Medical Microbiology and Hospital Epidemiology, Hannover Medical School, 30625 Hannover, Germany; 3 Chemical Biology Research Center, School of Pharmaceutical Sciences, Wenzhou Medical University, 325035 Wenzhou, China; 4 Department of Neuropathology, Faculty of Medicine and University Hospital Cologne, University of Cologne, 50931 Cologne, Germany; 5 Institute for Experimental Infection Research, TWINCORE Centre for Experimental and Clinical Infection Research, a joint venture between the Hannover Medical School and the Helmholtz Centre for Infection Research, 30625 Hannover, Germany; 6 Institute for Clinical Chemistry and Pathobiochemistry, Otto-von-Guericke University Magdeburg, 39120 Magdeburg, Germany; 7 Cluster of Excellence-Resolving Infection Susceptibility (RESIST), Hannover Medical School, 30625 Hannover, Germany; 8 Department of Molecular Biosciences, Stockholm University, 10691 Stockholm, Sweden; 9 Institute for Experimental Internal Medicine, Otto-von-Guericke University Magdeburg, 39120 Magdeburg, Germany

**Keywords:** OTUB1, dendritic cell, signal transduction, ubiquitination, innate immunity, Cell signalling, Mechanisms of disease, Innate immunity, Infection, Inflammation

## Abstract

Dendritic cells (DCs) are indispensable for defense against pathogens but may also contribute to immunopathology. Activation of DCs upon the sensing of pathogens by Toll-like receptors (TLRs) is largely mediated by pattern recognition receptor/nuclear factor-κB (NF-κB) signaling and depends on the appropriate ubiquitination of the respective signaling molecules. However, the ubiquitinating and deubiquitinating enzymes involved and their interactions are only incompletely understood. Here, we reveal that the deubiquitinase OTU domain, ubiquitin aldehyde binding 1 (OTUB1) is upregulated in DCs upon murine *Toxoplasma* *gondii* infection and lipopolysaccharide challenge. Stimulation of DCs with the TLR11/12 ligand *T. gondii* profilin and the TLR4 ligand lipopolysaccharide induced an increase in NF-κB activation in OTUB1-competent cells, resulting in elevated interleukin-6 (IL-6), IL-12, and tumor necrosis factor (TNF) production, which was also observed upon the specific stimulation of TLR2, TLR3, TLR7, and TLR9. Mechanistically, OTUB1 promoted NF-κB activity in DCs by K48-linked deubiquitination and stabilization of the E2-conjugating enzyme UBC13, resulting in increased K63-linked ubiquitination of IRAK1 (IL-1 receptor-associated kinase 1) and TRAF6 (TNF receptor-associated factor 6). Consequently, DC-specific deletion of OTUB1 impaired the production of cytokines, in particular IL-12, by DCs over the first 2 days of *T. gondii* infection, resulting in the diminished production of protective interferon-γ (IFN-γ) by natural killer cells, impaired control of parasite replication, and, finally, death from chronic *T.* *encephalitis*, all of which could be prevented by low-dose IL-12 treatment in the first 3 days of infection. In contrast, impaired OTUB1-deficient DC activation and cytokine production by OTUB1-deficient DCs protected mice from lipopolysaccharide-induced immunopathology. Collectively, these findings identify OTUB1 as a potent novel regulator of DCs during infectious and inflammatory diseases.

## Introduction

Dendritic cells (DCs) are key sentinel cells and professional antigen-presenting cells (APCs) of the immune system.^1^ They bridge innate and adaptive immune responses and play indispensable roles in host defense against invading pathogens, including viruses, bacteria, and parasites. Individual DC populations are adapted to their anatomical niche and particular function. Type 1 conventional DCs (cDC1s) and cDC2s develop in BATF3/IRF-8- and IRF4-dependent manners, respectively, from a common DC precursor, whereas plasmacytoid DCs (pDCs) may arise from common DCs and common lymphoid progenitors.^1,2^

The detection of pathogens by DCs is primarily mediated by pattern recognition receptors (PRRs), which are essential for DC activation and subsequent immune responses. Among PRRs are the Toll-like receptors (TLRs), which consist of 10 human and 12 murine members. Interestingly, TLR11 and TLR12, which are expressed in only mice, sense very few pathogen-associated molecular pattern molecules (PAMPs), including *Toxoplasma* (*T*.) *gondii* profilin (TgPFN), which activates the MyD88/nuclear factor-κB (NF-κB) pathway, leading to protective interleukin-12 (IL-12) production by CD8^+^ cDC1s within a few hours after infection.^3–5^ In contrast, TLR4 is expressed by many cell types in mice and humans, including cDC1s, cDC2s, and pDCs, and induces activation of the NF-κB and mitogen-activated protein kinase (MAPK) pathways upon engagement by Gram-negative bacterial lipopolysaccharides (LPS). Notably, exaggerated stimulation of TLR4 by LPS may lead to severe immunopathology, as observed in sepsis.^6^

The NF-κB pathway is tightly modulated by post-translational modifications (PTMs), including phosphorylation and ubiquitination. These PTMs are highly dynamic and reversible and thereby enable a swift and economical adaptation to environmental changes. Ubiquitination is a process involving the covalent fusion of one or more monomers of ubiquitin, an 8.5 kDa regulatory protein, to target proteins. Ubiquitin molecules can be linked via Met 1 (M1) or one of their seven Lys (K) residues, K6, K11, K27, K29, K33, K48, and K63, to form distinct polyubiquitin chains. Depending on the type of ubiquitination, proteins may be degraded or functionally altered, particularly during signal transduction. Ubiquitination of target proteins requires the concerted actions of E2-conjugating enzymes, which determine the type of ubiquitination, and E3 ubiquitin ligases, which provide substrate specificity.^7^ Activation of the NF-κB pathway is critically regulated by several E2-conjugating enzymes and E3 ligases, including UBC13, Pellino, and TRAF6 (TNF receptor-associated factor 6).^8–10^

Ubiquitination is reversible and can be counterregulated by a family of isopeptidases known as deubiquitinases (DUBs), which comprises ~100 members in mice and humans.^11^ DUBs have emerged as crucial regulators of the immune system via their reduction of ubiquitination in key signaling pathways in multiple immune cell populations, including DCs. We and others have identified the DUB A20 as a key controller of immune homeostasis and responses to PAMPs through its inhibition of NF-κB signaling in DCs.^12–14^

OTU domain, ubiquitin aldehyde binding 1 (OTUB1) is a DUB that preferentially cleaves K48-linked polyubiquitination, which is associated with protein degradation. Functionally, OTUB1 has been shown to regulate numerous signaling pathways by stabilizing signaling molecules such as p100,^15^ UBE2E1,^16^ Snail,^17^ DEPTOR,^18^ YB-1,^19^ SMAD2/3,^20^ c-IAP,^21^ p53,^22^ AKT,^23^ and SOCS1.^24^ However, the in vivo function of OTUB1 in the immune system, particularly that in DCs, is still unclear.

To study the DC-specific function of OTUB1, we generated CD11c-Cre OTUB1^fl/fl^ mice, which contained DCs deficient in OTUB1 and developed normally. However, due to a deficiency in the DC-specific production of the cytokines IL-12, IL-6, and tumor necrosis factor (TNF), CD11c-Cre OTUB1^fl/fl^ mice were more susceptible to *T. gondii* infection, but more resistant to LPS challenge. Mechanistically, OTUB1 promoted cytokine production in DCs by downregulating K48-linked ubiquitination and increasing the stability of UBC13, an E2-conjugating enzyme critical for the activation of canonical NF-κB and MAPK signaling. Thus, OTUB1 acts as a key regulator of NF-κB activity in DCs in infectious and inflammatory diseases.

## Results

### OTUB1 is upregulated in DCs during *T. gondii* infection and LPS challenge

Since OTUB1 has been reported to interact with molecules critical for activation of the immune system and DCs are key immune cells that protect the host from various infectious diseases, including toxoplasmosis,^25,26^ but also contribute to immunopathology in sepsis,^21,27–29^ we asked whether DC-specific OTUB1 is regulated during murine toxoplasmosis and LPS-induced sepsis.

**Fig. 1:**
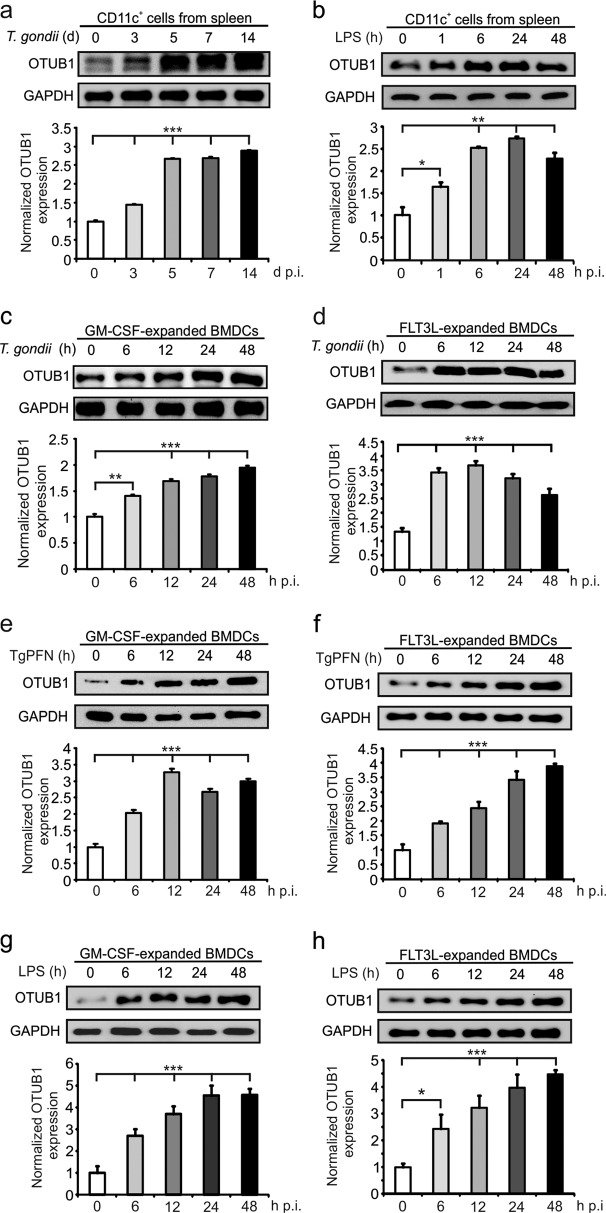
OTUB1 expression is upregulated in DCs by *T. gondii* infection and LPS stimulation. **a**, **b** Mice were infected i.p. with five *T. gondii* cysts (**a**) or challenged with LPS (**b**) for the indicated time periods. CD11c^+^ cells were isolated from spleens by magnetic sorting, and OTUB1 expression was measured by WB analysis (upper panels). The lower panels show the relative expression of OTUB1 (*n* = 3 for each group). **c**–**h** GM-CSF-expanded (**c**, **e**, and **g**) and FLT3L-expanded (**d**, **f**, and **h**) BMDCs generated from C57BL/6 mice were stimulated in vitro with tachyzoites at an MOI of 3 (**c**, **d**), 1 μg/ml TgPFN (**e**, **f**), or 500 ng/ml LPS (**g**, **h**) for the indicated time periods. OTUB1 expression in BMDCs was analyzed by WB analysis (upper panels). The lower panels show the relative expression of OTUB1 (*n* = 3 for each group). Data are displayed as the mean ± SD. *p < 0.05, **p < 0.01, and ***p < 0.001

The OTUB1 protein was constitutively expressed in splenic CD11c^+^ cells, which comprise all major populations of DCs,^1^ but its levels were significantly increased during *T. gondii* infection and LPS challenge (Fig. 1a, b). To substantiate these in vivo findings, in vitro-expanded bone marrow-derived DCs (BMDCs) were stimulated with *T. gondii*, which activates TLR2, TLR4, TLR7, and TLR11/12;^30^ TgPFN, a protein that signals via TLR11/12 and is crucial for parasite motility as well as host cell invasion and egress;^31^ or LPS, which binds TLR4, for the indicated time and analyzed by western blot (WB) for OTUB1 expression. Consistent with the in vivo data, all three stimuli significantly increased OTUB1 protein levels in in vitro-stimulated, granulocyte–macrophage colony-stimulating factor (GM-CSF)- and FMS-like tyrosine kinase 3 ligand (FLT3L)-expanded BMDCs (Fig. 1c–h). The upregulation of OTUB1 was a consequence of increased gene transcription, as indicated by increased OTUB1 messenger RNA (mRNA) levels after TgPFN- and LPS-induced stimulation (Supplementary Fig. 1a, b). The finding that OTUB1 is upregulated in *T. gondii*-infected, TgPFN- and LPS-stimulated DCs raises the question of whether OTUB1 is induced only upon stimulation of TLR/MyD88/NF-κB signaling or whether OTUB1 also acts as a feedback regulator of this signaling pathway.

### OTUB1 positively regulates proinflammatory NF-κB signaling in DCs

To determine the functional role of OTUB1 in DCs, we crossed CD11c-Cre mice^32^ with OTUB1^fl/fl^ mice^24^ to generate CD11c-Cre OTUB1^fl/fl^ mice. WB analysis of DCs, macrophages, natural killer (NK) cells, T cells, and B cells isolated from the spleens of OTUB1^fl/fl^ and CD11c-Cre OTUB1^fl/fl^ mice showed that OTUB1 had been specifically and efficiently deleted from the DCs of CD11c-Cre OTUB1^fl/fl^ mice (Supplementary Fig. 1c). In addition, OTUB1 expression was strongly reduced in in vitro-expanded BMDCs but not bone marrow-derived macrophages (BMDMs) (Supplementary Fig. 1d). The CD11c-Cre OTUB1^fl/fl^ mice were born at a normal Mendelian ratio, did not develop any clinical signs of disease, and exhibited a preserved, normal immune system, as revealed by flow cytometry (Supplementary Fig. 1e–i). Consistent with the finding that OTUB1 had no impact on DC differentiation in vivo (Supplementary Fig. 1f), OTUB1 did not interfere with the in vitro development of FLT3L- and GM-CSF-expanded BMDC subpopulations, including CD8^+^ CD11c^+^ cDC1, CD11b^+^ CD11c^+^ cDC2, and PDCA1^+^ CD11c^+^ pDCs (Supplementary Fig. 1j).

**Fig. 2:**
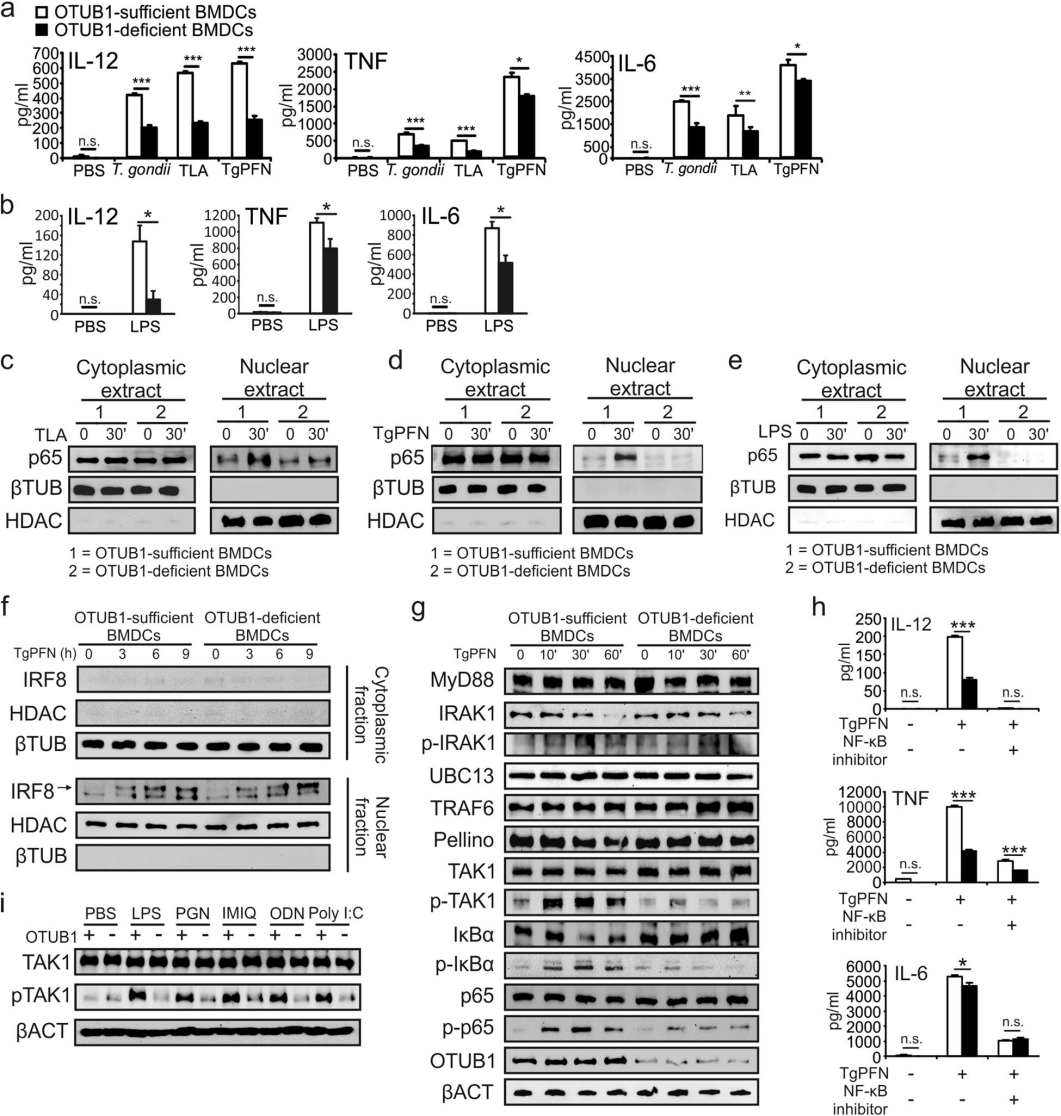
OTUB1 deletion in DCs impairs the cytokine response during *T. gondii* infection and LPS stimulation by reducing NF-κB activation. **a**, **b** OTUB1-sufficient and OTUB1-deficient BMDCs were left untreated or stimulated with *T. gondii* tachyzoites (MOI = 3), different antigens (TLA and TgPFN) or LPS for 24 h. Concentrations of cytokines in the supernatant were analyzed by ELISA (*n* = 4). (**c**–**e**) FLT3L-expanded BMDCs were left untreated or stimulated with TLA (**c**), TgPFN (**d**), or LPS (**e**) for 30 min. Levels of p65 in the cytoplasmic and nuclear fractions were analyzed by WB analysis. **f** FLT3L-expanded BMDCs were stimulated with TgPFN for the indicated time periods. IRF8 in the cytoplasmic and nuclear extracts was detected by WB analysis. **g** FLT3L-expanded BMDCs were stimulated with TgPFN for the indicated time periods. Whole-cell lysates were analyzed by WB analysis with the indicated antibodies. **h** FLT3L-expanded BMDCs were left untreated or stimulated with TgPFN in the presence or absence of NF-κB inhibitor for 24 h. Cytokine levels in the supernatants of cell cultures were measured by ELISA (*n* = 4). **i** FLT3L-expanded BMDCs were stimulated with LPS, PGN, IMIQ, ODN, and poly I:C for 30 min. Whole-cell lysates were analyzed by WB analysis with the indicated antibodies. Data are shown as the mean ± SD. *p < 0.05, **p < 0.01, and ***p < 0.001; n.s. not significant

To clarify the role of OTUB1 in DCs after TLR engagement, we stimulated in vitro FLT3L-expanded BMDCs with different TLR ligands. As shown in Fig. 2a, b, OTUB1-deficient BMDCs produced significantly less IL-12, TNF, and IL-6 after stimulation with *T. gondii*, *T. gondii* lysate antigen (TLA), TgPFN (which all signal via MyD88^4^) (Fig. 2a), and LPS (Fig. 2b). The reduced cytokine production in OTUB1-deficient BMDCs was also confirmed by measurement of the mRNA expression of IL-12α, IL-12β, TNF, and IL-6 after TgPFN- and LPS-induced stimulation by quantitative PCR (Supplementary Fig. 2a, b). In addition, engagement of TLR2, TLR7, and TLR9, which all signal via MyD88, and TLR3, which signals in a MyD88-independent manner through TRIF,^33^ resulted in significantly reduced IL-12, TNF, and IL-6 production by OTUB1-deficient BMDCs (Supplementary Fig. 2c), illustrating the stimulatory role of OTUB1 in proinflammatory TLR signaling.

To understand how OTUB1 promotes cytokine production in DCs, we stimulated FLT3L-expanded BMDCs with TLA (Fig. 2c), TgPFN (Fig. 2d), and LPS (Fig. 2e) and analyzed signaling molecules by WB analysis. WB analysis of the cytoplasmic and nuclear fractions of untreated and stimulated OTUB1-sufficient and OTUB1-deficient BMDCs revealed that after 30 min of stimulation, the nuclear translocation of NF-κB p65 in OTUB1-deficient BMDCs was reduced (Fig. 2c–e), suggesting that OTUB1 augments NF-κB activation. In murine DCs, the recognition of TgPFN by TLR11 and TLR12 induces IL-12 production by activating two signaling pathways: the NF-κB and IRF8 pathways.^5,34^ In contrast to NF-κB accumulation, stimulation of BMDCs with TgPFN resulted in the equivalent nuclear accumulation of IRF8 in BMDCs of both genotypes, indicating that OTUB1 regulates only NF-κB signaling (Fig. 2f). Next, we analyzed MyD88-mediated signaling upstream of p65. Reduced phosphorylation of TAK1, IκBα, and p65 was detected in OTUB1-deficient BMDCs upon stimulation with TgPFN (Fig. 2g), suggesting that OTUB1 is required for the full-fledged activation of NF-κB. To reinforce the assertion that OTUB1 promotes IL-12 production by increasing NF-κB activity, we studied the effect of IKK inhibition on TgPFN-induced cytokine production in OTUB1-sufficient and OTUB1-deficient BMDCs. Abolishment of NF-κB activity by the inhibitor dramatically reduced IL-12 production by both OTUB1-sufficient and OTUB1-deficient BMDCs and blunted the difference in IL-12 production between the two cell populations (Fig. 2h).

Since the phosphorylation of TAK1 is a crucial step for activation of the NF-κB pathway in TLR-initiated signaling pathways,^35^ we assessed the phosphorylation of TAK1 following the stimulation of TLR2, TLR3, TLR4, TLR7, and TLR9 with their specific ligands. In good agreement with the increased TAK1 phosphorylation in OTUB1-competent BMDCs upon TLR11/12 stimulation (Fig. 2g), the phosphorylation of TAK1 was also strongly increased in OTUB1-competent BMDCs upon the engagement of TLR2, TLR4, TLR7, TLR9, and TLR3 (Fig. 2i).

Stimulation of TLR3 activates NF-κB by RIP1-mediated TAK1 phosphorylation.^36^ Therefore, we further explored whether increased MyD88-independent TLR3/TRIF-mediated activation of TAK1 in OTUB1-competent BMDCs also resulted in the enhanced activation of NF-κB. In fact, stimulation with polyinosinic-polycytidylic acid (poly I:C) augmented IκBα and p65 phosphorylation in OTUB1-competent DCs compared to OTUB1-deficient DCs (Supplementary Fig. 2d). Likewise, stimulation with TNF, which also induces RIP1-dependent TAK1 activation,^37^ or IL-1β, which activates TAK1 via MyD88 and TRAF6,^38^ increased the phosphorylation of IκBα and p65 in OTUB1-competent BMDCs (Supplementary Fig. 2e, f, respectively), further illustrating that OTUB1 supports NF-κB activation in DCs upon their stimulation with both MyD88-dependent and MyD88-independent proinflammatory stimuli.

In addition to the activation of NF-κB, the engagement of TLR4 and TLR11/12 resulted in activation of the MAPKs p38, extracellular signal-regulated kinase 1/2 (ERK1/2), and c-Jun N-terminal kinase (JNK), which induce the expression of genes associated with innate immunity, inflammation, and cell survival.^39^ The NF-κB and MAPK pathways cross-regulate each other and share some signaling molecules, including TAK1, UBC13, and TRAFs.^35,40,41^ Therefore, we studied the impact of OTUB1 on MAPK activation upon TLR11/12 and TLR4 stimulation. Stimulation of OTUB1-competent BMDCs with both TgPFN and LPS resulted in increased p38, ERK1/2, and JNK phosphorylation compared to that in OTUB1-deficient BMDCs (Supplementary Fig. 2g, h), demonstrating that OTUB1 augments the activation of both NF-κB and MAPKs upon the engagement of TLR4 and TLR11/12.

### OTUB1 regulates NF-κB activity via deubiquitinating and stabilizing UBC13

**Fig. 3:**
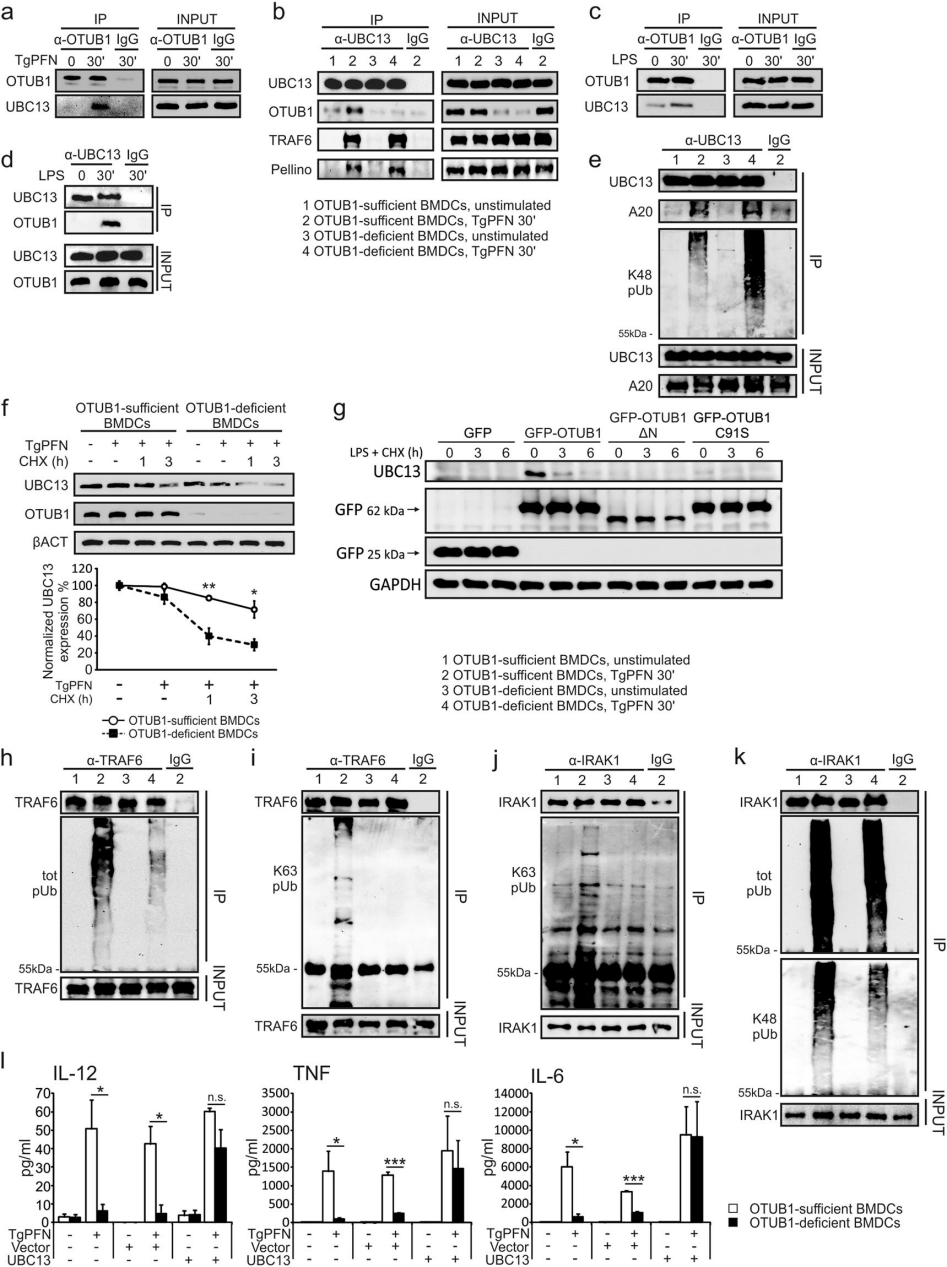
OTUB1 stabilizes UBC13 by reducing its K48-linked polyubiquitination. **a**–**e** Cytoplasmic proteins from unstimulated, TgPFN-stimulated (**a**, **b**, **e**) and LPS-stimulated (**c**, **d**) BMDCs were immunoprecipitated with the indicated antibodies. Immunoprecipitates and input were analyzed by WB analysis with the indicated antibodies. **f** OTUB1-sufficient and OTUB1-deficient FLT3L-expanded BMDCs were pretreated with TgPFN (1 μg/ml) for 1 h or left unstimulated. Then, cycloheximide (CHX, 100 μg/ml) was added for the indicated time period. Protein levels of UBC13 in whole-cell lysates were analyzed by WB analysis (upper panel). The lower panel shows the relative levels of UBC13 normalized to βACT levels (*n* = 3). **g** NIH 3T3 cells were transfected with OTUB1 siRNA for 36 h. Then, the cells were transfected with GFP, GFP-OTUB1, GFP-OTUB1 ΔN, or GFP-OTUB1 C91S plasmids. After 24 h, the cells were treated with CHX + LPS for 0, 3, and 6 h. Whole-cell lysates were then isolated and analyzed with the indicated antibodies. **h**–**k** GM-CSF-expanded BMDCs were left unstimulated or stimulated with TgPFN in the presence of MG132. Cytoplasmic proteins were isolated and immunoprecipitated with anti-TRAF6 (**h**, **i**) and anti-IRAK1 (**j**, **k**) antibodies. Immunoprecipitates and input were analyzed with the indicated antibodies. **l** FLT3L-expanded BMDCs were left untreated or transduced with UBC13-expressing lentivirus or vector lentivirus for 72 h, followed by stimulation with TgPFN for 24 h. Cytokines in the supernatants of cell cultures were measured by flow cytometry (*n* = 4). Data are displayed as the mean ± SD (**d**) or mean ± SD (**g**). *p < 0.05, **p < 0.01, and ***p < 0.001; n.s. not significant

To investigate the mechanism by which OTUB1 promotes NF-κB activation, we screened for OTUB1-interacting signaling molecules in the NF-κB pathway by immunoprecipitation (IP) and WB analysis. In the screen, UBC13 (UBE2N) emerged as a promising target of OTUB1 because it was coimmunoprecipitated with OTUB1 upon stimulation with TgPFN (Fig. 3a, b) and LPS (Fig. 3c, d), and a previous study reported the interaction between nuclear OTUB1 and UBC13 in the context of DNA double-strand breaks.^42^ Functionally, UBC13 acts as an E2-conjugating enzyme in the NF-κB pathway. In cooperation with the E3 ligase Pellino, UBC13 can add K63-linked polyubiquitin chains to phosphorylated IRAK1 (IL-1 receptor-associated kinase 1), which enables IRAK1 to directly activate NF-κB essential modulator (NEMO).^43^ Additionally, phosphorylated IRAK1 that has not been ubiquitinated induces the recruitment of TRAF6, which undergoes subsequent K63-linked ubiquitination by itself to activate NEMO through TAK1. In this process, the E2 activity of UBC13 is indispensable for the self-ubiquitination and activation of TRAF6. Therefore, UBC13 can positively regulate NF-κB signaling via ubiquitinating IRAK1 and TRAF6. In good agreement with these findings, we detected the interactions of UBC13 with TRAF6 and Pellino in TgPFN-stimulated BMDCs (Fig. 3b). In contrast, we did not detect any interactions between OTUB1 and IRAK1, Pellino, or TRAF6 (Supplementary Fig. 3a). It has been reported that UBC13 protein stability is controlled by the E3 ligase A20, which adds K48-linked polyubiquitin chains to UBC13 to induce its proteasomal degradation.^44^ Since (i) OTUB1 interacts with UBC13 (Fig. 3a–d), (ii) UBC13 can undergo K48-linked ubiquitination,^44^ and (iii) OTUB1 is a K48-specific DUB,^45^ we explored the effect of OTUB1 on the status of K48-linked UBC13 ubiquitination. As shown in Fig. 3e, OTUB1 reduced the K48-linked ubiquitination of UBC13 in TgPFN-stimulated BMDCs. This reduced K48-linked ubiquitination of UBC13 is due to the deubiquitinating activity of OTUB1 against UBC13, but not an interaction between OTUB1 and A20 (Supplementary Fig. 3b), the E3 ligase that mediates K48-linked ubiquitination of UBC13.^44^ Since K48-linked ubiquitination is associated with protein degradation, we studied the impact of OTUB1 on UBC13 protein stability using a cycloheximide (CHX) chase assay. Consistent with the results shown in Fig. 3e, the decreased K48-linked ubiquitination of UBC13 in the presence of OTUB1 resulted in reduced protein degradation (Fig. 3f). Notably, the influence of OTUB1 on UBC13 was restricted to only PTM and protein degradation, as the mRNA expression of UBC13 was independent of OTUB1 (Supplementary Fig. 3c). In addition, accelerated degradation of UBC13 was observed in OTUB1 small interfering RNA (siRNA)-treated NIH 3T3 cells (Supplementary Fig. 3d), ruling out the possibility that the enhanced UBC13 degradation in OTUB1-deficient BMDCs was cell type specific or due to the genotoxic effect of the CD11c-Cre transgene. In contrast to UBC13, the protein stability of Uev1a, the noncatalytic E2-like partner protein of UBC13,^46–50^ in TgPFN-stimulated BMDCs was independent of OTUB1 (Supplementary Fig. 3e). Additionally, stimulation of OTUB1-competent and OTUB1-deficient BMDCs with TgPFN and LPS did not reduce the amount of cIAP1 protein within 60 min (Supplementary Fig. 3f), indicating that the swift activation of MyD88-dependent TLR signaling does not impact OTUB1-dependent cIAP1 stability, as observed upon stimulation with TWEAK.^21^

OTUB1 can reduce the ubiquitination of substrates by two mutually nonexclusive mechanisms. First, OTUB1 can cleave polyubiquitin chains from substrates by its catalytic activity, which is mainly dependent on the C91 residue and shows specificity for K48-linked polyubiquitin chains. Second, OTUB1 can prevent the K48- and K63-linked polyubiquitination of substrates by binding the corresponding E2/E3 complex and blocking ubiquitin transfer by its N-terminal ubiquitin-binding domain.^16,45,51^ To clarify which mechanism is responsible for the deubiquitination and stabilization of UBC13, we transfected NIH 3T3 fibroblasts, in which OTUB1 had been suppressed via siRNA, with plasmids encoding (i) a catalytically inactive OTUB1-C91S-GFP mutant; (ii) an OTUB1-ΔN-GFP mutant lacking the N-terminal ubiquitin-binding domain, which is responsible for the noncatalytic inhibition of substrate ubiquitination; and (iii) wild-type OTUB1-GFP. The expression of UBC13 in wild-type OTUB1-transfected NIH 3T3 cells was highly preserved, and after 3 h of stimulation with LPS in combination with CHX, UBC13 expression was still detectable (Fig. 3g). However, UBC13 was undetectable in NIH 3T3 fibroblasts transfected with the OTUB1-C91S-GFP and OTUB1-ΔN-GFP mutants, illustrating that both the C91 residue and the N-terminus of OTUB1 play a critical role in the stabilization of UBC13.

Since UBC13 was stabilized by reduced K48-linked ubiquitination in OTUB1-competent BMDCs, we inspected the impact of OTUB1 on the ubiquitination status of IRAK1 and TRAF6. In response to TgPFN, total and K63-ubiquitinated IRAK1 and TRAF6 were increased in the presence of OTUB1 (Fig. 3h–k). In contrast, K48-linked polyubiquitination of TRAF6 was only weakly induced by TgPFN in OTUB1-competent and OTUB1-deficient BMDCs (Supplementary Fig. 3g). Interestingly, K48-linked polyubiquitination of IRAK1 was also increased in OTUB1-competent BMDCs (Fig. 3k), which is consistent with sequential K63- and K48-linked polyubiquitination of IRAK1 in TLR-induced MyD88-dependent NF-κB activation.^43,52^

To directly verify that the reduced production of proinflammatory cytokines in OTUB1-deficient BMDCs was a consequence of the rapid degradation of UBC13, we overexpressed UBC13 with lentivirus (Supplementary Fig. 3h). As shown in Fig. 3l, untransduced and mock-transduced OTUB1-deficient BMDCs produced significantly lower amounts of IL-12, TNF, and IL-6 than OTUB1-sufficient BMDCs, whereas OTUB1-deficient BMDCs transduced with UBC13 produced amounts of proinflammatory cytokines similar to those in UBC13-transduced OTUB1-sufficient BMDCs (Fig. 3l). This demonstrates that the restoration of UBC13 corrected the defect in cytokine production in OTUB1-deficient cells. Taken together, these results indicate that OTUB1 promotes *T. gondii*-induced NF-κB activation and cytokine production in DCs by directly deubiquitinating and stabilizing the E2 enzyme UBC13. Ubiquitinated TRAF6 induces the activation of its downstream kinase TAK1, which is essential for NF-κB and MAPK activity. In addition to TRAF6, TAK1 can be directly activated by unanchored ubiquitin chains.^53^ Unanchored ubiquitin chains can originate from the DUB-dependent release of ubiquitin from substrates during proteasomal degradation or can be synthesized de novo using monomeric ubiquitin as a substrate.^54^ Since OTUB1 is a DUB and UBC13 in cooperation with Uev1a and TRAF6 mediates the formation of unanchored ubiquitin,^49,53,55,56^ we hypothesized that unanchored ubiquitin would be increased in TgPFN-activated OTUB1-competent DCs. We performed a free ubiquitin assay^57^ and discovered that 30 min after TgPFN stimulation, the accumulation of unanchored ubiquitin monomers, dimers, and polyubiquitin chains was strongly increased in OTUB1-sufficient BMDCs compared to OTUB1-deficient BMDCs (Supplementary Fig. 3i). Thus, in OTUB1-competent BMDCs, the increased amount of free ubiquitin may further contribute to the activation of TAK1 and increased NF-κB activation.

### OTUB1 is required for high levels of cytokine production by CD11c^+^ cells and subsequent IFN-γ responses in early toxoplasmosis

**Fig. 4:**
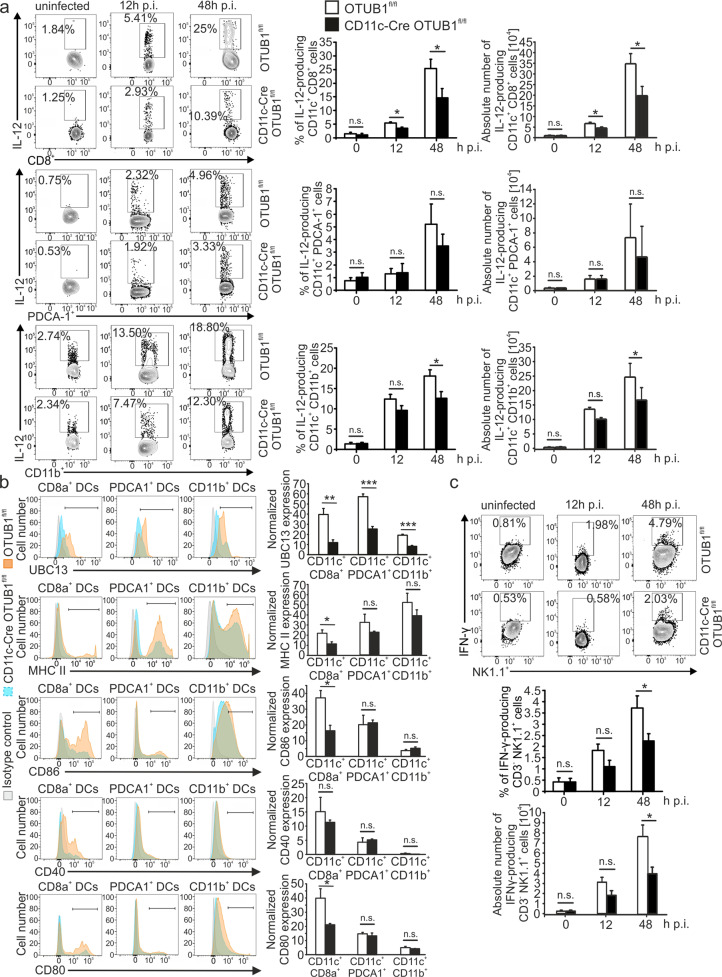
OTUB1 deletion in DC impairs the cytokine response during *T. gondii* infection. **a**–**c** OTUB1^fl/fl^ and CD11c-Cre OTUB1^fl/fl^ mice were infected i.p. with 50,000 tachyzoites for 12 and 48 h, respectively. Peritoneal cells were collected by lavage of the peritoneal cavity and analyzed by flow cytometry to detect IL-12 production in CD11c^+^ CD8a^+^, CD11c^+^ PDCA-1^+^, and CD11c^+^ CD11b^+^ DCs (**a**); UBC13, MHC II, CD40, CD86 and CD80 expression in CD11c^+^ CD8a^+^, CD11c^+^ PDCA-1^+^, and CD11c^+^ CD11b^+^ DCs (**b**); and IFN-γ production in NK cells (**c**). Representative flow cytometry plots (left panels) and statistics (right panels) are shown (*n* = 8). Data are shown as the mean ± SD. *p < 0.05, **p < 0.01, and ***p < 0.001; n.s. not significant

To determine the functional role of OTUB1 in DCs in vivo, we induced toxoplasmosis in CD11c-Cre OTUB1^fl/fl^ and control mice. CD8^+^ cDC1s are the main source of IL-12 within a few hours after infection,^26,58^ and shortly thereafter, interferon-γ (IFN-γ)-primed CD11b^+^ cDC2s and PDCA1^+^ pDCs additionally contribute to IL-12 production.^34^ Therefore, we explored the impact of OTUB1 on DC-mediated IL-12 production during *T. gondii* infection. CD11c-Cre OTUB1^fl/fl^ and OTUB1^fl/fl^ control mice were i.p. infected with 50,000 tachyzoites expressing GFP (PTG-GFP strain, type II strain), and cells in the peritoneal cavity were collected at 12 and 24 h postinfection (p.i.) for further analysis. In good agreement with the OTUB1-dependent cytokine production observed in vitro (Fig. 2a), the CD8^+^ cDC1s of CD11c-Cre OTUB1^fl/fl^ mice showed a significantly reduced percentage and absolute number of IL-12-producing DCs in comparison to OTUB1^fl/fl^ mice at 12 and 48 h p.i. (Fig. 4a). Additionally, the CD11b^+^ cDC2s of CD11c-Cre OTUB1^fl/fl^ mice exhibited reduced IL-12 production 48 h, but not 12 h p.i. (Fig. 4a). In contrast, IL-12 production by F4/80^+^ macrophages was identical in both mouse strains (Supplementary Fig. 4a). Additionally, the production of TNF and IL-6 was significantly reduced in all DC subtypes of CD11c-Cre OTUB1^fl/fl^ mice at 2 days p.i. (Supplementary Fig. 4b, c). In good agreement with the UBC13-stabilizing function of OTUB1 in TgPFN-stimulated BMDCs (Fig. 3f), we detected the increased expression of UBC13 in CD8^+^ cDC1s, PDCA1^+^ DCs, and CD11b^+^ cDC2s isolated from OTUB1^fl/fl^ mice after infection with *T. gondii* (Fig. 4b, first top panel), confirming the critical role of OTUB1 in stabilizing UBC13 in DCs in vivo. In addition to the induction of cytokines, NF-κB activity drives the expression of immunologically important cell surface molecules, such as MHC (major histocompatibility complex) class II, CD40, CD80, and CD86, which regulate the interaction of DCs with T cells.^59^ Here, we observed significantly reduced expression of MHC II, CD80, and CD86 in the CD8^+^ cDC1s of CD11c-Cre OTUB1^fl/fl^ mice 48 h p.i. (Fig. 4b), substantiating the role of OTUB1 in NF-κB activation, particularly that in this DC subtype.

Given that CD8^+^ cDC1-derived IL-12 is indispensable for the initiation of a potent IFN-γ response in NK cells upon *T. gondii* infection,^60–62^ the reduction in early IL-12 production in the CD8^+^ cDC1s of CD11c-Cre OTUB1^fl/fl^ mice was accompanied by a reduced IFN-γ response in NK cells (Fig. 4c), but not in CD4^+^ and CD8^+^ T cells (Supplementary Fig. 4d). The strong OTUB1-dependent reduction in IL-12 production by CD11b^+^ cDC2s at 48 h p.i. is consistent with the observation that IFN-γ-mediated priming of these DC populations is required for their TLR11/12-dependent production of IL-12.^5,63^ The impaired immune response of OTUB1-deficient DCs resulted in a reduction in serum IL-12 and IL-6, and, to a lesser extent, TNF at 48 h p.i. (Supplementary Fig. 4e).

Together, these data suggest a pivotal role for OTUB1 in driving NF-κB-dependent immune reactions in DCs, including IL-12 production, which is critical for a potent NK cell-dependent IFN-γ response in toxoplasmosis.^62^

### CD11c-Cre OTUB1^fl/fl^ mice fail to control expansion of *T. gondii*

**Fig. 5:**
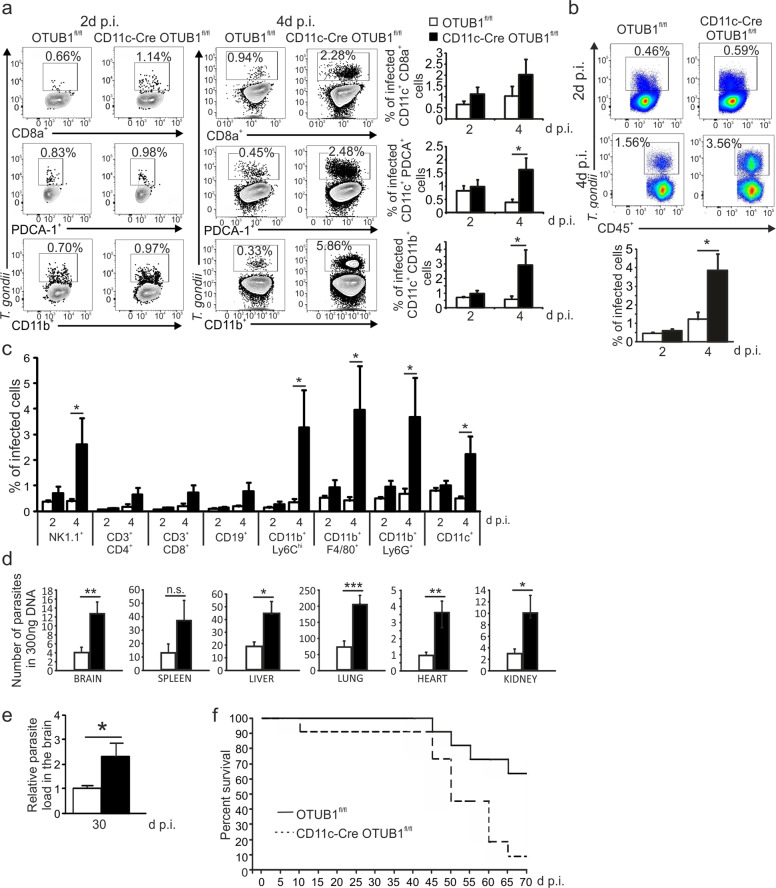
OTUB1 deletion in DCs leads to impaired parasite control in toxoplasmosis. **a**–**c** OTUB1^fl/fl^ and CD11c-Cre OTUB1^fl/fl^ mice were infected i.p. with 50,000 tachyzoites. Peritoneal cells were isolated from the peritoneal cavities and analyzed by flow cytometry at 2 and 4 days p.i. **a** Percentages of infected CD11c^+^ CD8a^+^, CD11c^+^ PDCA-1^+^, and CD11c^+^ CD11b^+^ DCs. **b** Percentages of infected peritoneal CD45^+^ cells in OTUB1^fl/fl^ and CD11c-Cre OTUB1^fl/fl^ mice. **c** Percentages of infected peritoneal leukocyte subpopulations in OTUB1^fl/fl^ and CD11c-Cre OTUB1^fl/fl^ mice. Representative FACS plots (upper panel) and statistics (lower panel) are shown (*n* = 10 for each group). **d** Mice were infected i.p. with 50,000 tachyzoites for 10 days. Parasite loads in different organs were determined by semiquantitative PCR of tissue DNA (*n* = 5 for each group). **e**, **f** Mice were infected i.p. with three cysts. Parasite load in the brain 30 days p.i. was determined by semiquantitative PCR of tissue DNA (*n* = 4 for each group) (**e**). Survival was monitored for 70 days p.i. (**f**) (*n* = 10 for each group). Data are expressed as the mean ± SD. *p < 0.05, **p < 0.01, and ***p < 0.001; n.s. not significant

To investigate whether the diminished immune responses of OTUB1-deficient DCs affected parasite control, we i.p. infected the mice with 50,000 GFP-expressing tachyzoites and determined the composition of leukocytes and parasite load in the peritoneal cavity at 2 and 4 days p.i. The percentages of *T. gondii*-infected cDC2s, pDCs, and, to a lesser extent, CD8^+^ cDC1s were increased in CD11c-Cre OTUB1^fl/fl^ mice at 4 days p.i. (Fig. 5a). The number of leukocytes present at the site of infection was comparable between the two mouse strains (Supplementary Fig. 5a), but the intracellular parasite loads of the total CD45^+^ leukocytes in the peritoneal cavity (Fig. 5b) and the individual leukocyte subpopulations NK1.1^+^ NK cells, CD11b^+^ Ly6C^hi^ inflammatory monocytes, CD11b^+^ F4/80^+^ macrophages, and CD11b^+^ Ly6G^+^ neutrophils (Fig. 5c) were significantly increased in CD11c-Cre OTUB1^fl/fl^ mice. This suggests that the early defect in immune activation of OTUB1-deficient DCs resulted in insufficient control of the parasite at the early stage of infection. The massive release of *T. gondii* from infected cells may augment parasite dissemination to adjacent cells, and, subsequently, to other organs. Indeed, quantitative analysis of *T. gondii* genomic DNA revealed that multiple organs of CD11c-Cre OTUB1^fl/fl^ mice harbored significantly higher parasite loads than those of OTUB1^fl/fl^ control mice at day 10 p.i. (Fig. 5d).

Interestingly, in vitro infection with *T. gondii* resulted in similar parasite burdens in OTUB1-sufficient and OTUB1-deficient BMDCs with or without IFN-γ prestimulation, suggesting that OTUB1 does not interfere with the cell-intrinsic parasite killing mechanism in DCs (Supplementary Fig. 5b). Consistently, the production of the antiparasitic effector molecules inducible nitric oxide synthase (iNOS), IFN-γ-induced GTPase (IGPT), and guanylate-binding proteins (GBPs) was comparable between OTUB1-sufficient and OTUB1-deficient BMDCs (Supplementary Fig. 5c). *Toxoplasma gondii* promotes its systemic dissemination by hijacking the migratory properties of parasitized DCs.^64–66^ To address whether OTUB1 has an impact on *Toxoplasma*-induced hypermigration of DCs, we performed motility analyses. Upon challenge with *T. gondii*, parasitized OTUB1-sufficient and OTUB1-deficient BMDCs exhibited similar elevated velocities, and unchallenged DCs exhibited a nonsignificant difference in baseline velocity (Supplementary Fig. 5d). Thus, *T. gondii*-induced DC hypermigration was OTUB1 independent. Collectively, these data suggest that OTUB1 has no impact on DC-intrinsic parasite control or DC-mediated *T. gondii* dissemination.

Hou et al. ^61^ demonstrated that impaired IL-12 production by CD8^+^ cDC1s in acute toxoplasmosis resulted in a more severe course of chronic *Toxoplasma* encephalitis (TE) with an increased intracerebral parasite load. To address whether DC-specific OTUB1 regulates the course of chronic TE, we infected CD11c-Cre OTUB1^fl/fl^ mice and control mice with three brain cysts of the type II DX strain. At day 30 p.i., the intracerebral parasite load in CD11c-Cre OTUB1^fl/fl^ mice was significantly higher than that in control mice (Fig. 5e). In addition, the mortality of CD11c-Cre OTUB1^fl/fl^ mice during chronic TE was significantly increased (Fig. 5f). Notably, the intracerebral immune response of CD11c-Cre OTUB1^fl/fl^ mice was not impaired, as illustrated by the normal number of the intracerebral leukocyte populations DCs and T cells at day 30 p.i. (Supplementary Fig. 5e–g). Additionally, IL-12 production by DCs and IFN-γ production by CD4^+^ and CD8^+^ T cells were equal in mice of both genotypes (Supplementary Fig. 5h, i, respectively). In parallel to the increased intracerebral load, even the production of antiparasitic IGTP and iNOS as well as IL-1β and IL-18 was increased in CD11c-Cre OTUB1^fl/fl^ mice (Supplementary Fig. 5j), further indicating that the aggravated course of TE in CD11c-Cre OTUB1^fl/fl^ mice was caused by an impaired DC immune response early after infection but not by an insufficient intracerebral immune response.

### Supplementation with IL-12 restored the ability of CD11c-Cre OTUB1^fl/fl^ mice to control *T. gondii*

**Fig. 6:**
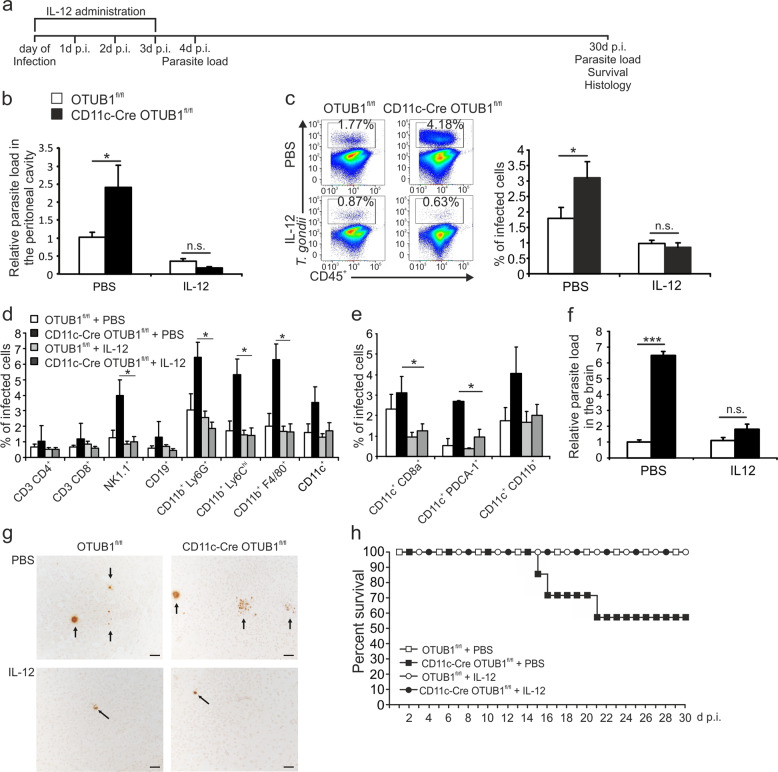
IL-12 supplementation rescues the defect in parasite control in CD11c-Cre OTUB1^fl/fl^ mice. **a** Experimental design scheme of IL-12 administration and analysis. Mice were i.p. injected with either PBS or 150 ng IL-12 daily from 0 to 3 days p.i. **b** The parasite load in peritoneal cells was determined by semiquantitative PCR of tissue DNA after infection with 50,000 tachyzoites for 4 days (*n* = 8 for each group). **c** Percentages of infected peritoneal CD45^+^ cells were analyzed by flow cytometry at day 4 after infection with 50,000 tachyzoites. Representative flow cytometry plots (left panel) and statistics (right panel) are shown (*n* = 8 for each group). **d**, **e** Percentages of infected peritoneal leukocyte subpopulations (**d**) and DC subsets (**e**) were analyzed by flow cytometry at day 4 after infection with 50,000 tachyzoites (*n* = 8 for each group). **f** Mice were i.p. infected with five cysts of the DX strain. At day 30 p.i., the parasite loads in the brains of surviving mice were determined by semiquantitative PCR. Two independent experiments with comparable results were performed. One representative experiment is shown (*n* = 4 for each group). **g** Intracerebral parasites in the brains of *T. gondii*-infected OTUB1^fl/fl^ and CD11c-Cre OTUB1^fl/fl^ mice at 30 days p.i. A PBS-treated OTUB1^fl/fl^ mouse (top left) shows a single focus consisting of a few *T. gondii* cysts and some tachyzoites in the white matter of the frontal lobe. The brain of a PBS-treated CD11c-Cre OTUB1^fl/fl^ mouse (top right) shows an increased number of parasitic foci (arrows) with *T. gondii* cysts and tachyzoites in the white matter of the frontal lobe. In both an OTUB1^fl/fl^ and a CD11c-Cre OTUB1^fl/fl^ mouse, IL-12 application reduced the intracerebral parasitic load, with only single cysts scattered throughout the frontal lobe (arrows). Immunohistochemistry with polyclonal rabbit anti-*T. gondii* (BioGenex, Fremont, CA, USA) and slight counterstaining with hemalum; original magnification ×200; scale bar (**a**–**d**): 50 µm. The photomicrographs shown are representative of three mice per experimental group. Similar results were obtained in a second independent experiment. **h** The survival of the mice was monitored daily up to 30 days after infection with cysts of strain 5 DX (*n* = 8 for each group). Data are displayed as the mean ± SD. *p < 0.05, and ***p < 0.001; n.s. not significant

Mice selectively lacking CD8^+^ cDC1s are extremely susceptible to toxoplasmosis due to impaired IL-12 and subsequent IFN-γ production.^26^ Treatment of these *Batf3*^−/−^ mice with 0.5 μg of IL-12 at days 0, 1, 2, 3 and 4 after infection restored resistance, illustrating that IL-12 production by CD8^+^ DCs is of central importance and independent of all other protective immune reactions in this cell population.^26^ To study whether the increased parasite expansion and dissemination in CD11c-Cre OTUB1^fl/fl^ mice were caused by the early defect in IL-12 production by DCs, we administered 150 ng of IL-12 i.p. to CD11c-Cre OTUB1^fl/fl^ and control mice at days 0, 1, 2, and 3 after infection, as shown in Fig. 6a. Here, although the defect in the production of cytokines by DCs was not limited to only IL-12 but also extended to TNF and IL-6, administration of IL-12 alone significantly reduced the *T. gondii* load in both CD11c-Cre OTUB1^fl/fl^ and control mice and abolished the difference in parasite load between mice of the two genotypes at 4 days p.i. (Fig. 6b–e), indicating that the early deficit in IL-12 production by DCs is responsible for the uncontrolled parasite expansion in acute infection. Interestingly, IL-12 administration in acute toxoplasmosis was also able to dramatically reduce the intracerebral parasite load in the brains of chronically infected CD11c-Cre OTUB1^fl/fl^ and OTUB1^fl/fl^ mice, and importantly, the intracerebral parasite load did not differ between mice of the two genotypes (Fig. 6f). Histopathology confirmed that IL-12 treatment strongly reduced parasite numbers and abolished differences between the two mouse strains (Fig. 6g). Consistent with the finding that a higher brain parasite load is associated with increased mortality (Fig. 5e, f), supplementation with IL-12 dramatically increased the survival of CD11c-Cre OTUB1^fl/fl^ mice (Fig. 6h). This suggests that the higher parasite burden and mortality of CD11c-Cre OTUB1^fl/fl^ mice during chronic infection are caused by a failure to restrict parasite expansion in the early stage via innate immunity. Collectively, these results suggest that DC-specific OTUB1 contributes to parasite control in both acute and chronic toxoplasmosis by facilitating early IL-12 production in DCs, which is indispensable for eliciting the protective IFN-γ response.

### OTUB1 regulates cytokine production in LPS-induced sepsis and death

**Fig. 7:**
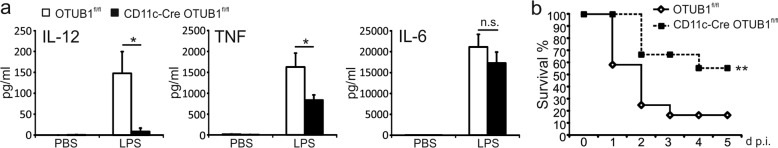
OTUB1 deficiency decreases LPS-induced mortality by reducing cytokine production. **a** OTUB1^fl/fl^ and CD11c-Cre OTUB1^fl/fl^ mice were i.p. administered 10 mg/kg LPS. After 3 h, blood was collected, and cytokines in the sera were analyzed by ELISA (*n* = 9). **b** OTUB1^fl/fl^ and CD11c-Cre OTUB1^fl/fl^ mice were i.p. administered a low dose of LPS (10 mg/kg), and survival was monitored daily for 6 days (*n* = 10). Data are displayed as the mean ± SD. *p < 0.05 and **p < 0.01; n.s. not significant

As shown in Fig. 1b, g, in addition to TgPFN stimulation, LPS stimulation upregulated OTUB1 expression in vivo and in vitro (Fig. 1b, g, h). Additionally, OTUB1 amplified NF-κB activation in LPS-stimulated BMDCs (Fig. 2e, i) and interacted with UBC13 (Fig. 3c, d), showing that OTUB1 also augments LPS-induced signaling and cellular responses. To investigate the functional role of DC-specific OTUB1 in LPS challenge, we injected lethal doses of LPS into CD11c-Cre OTUB1^fl/fl^ and OTUB1^fl/fl^ mice. Compared with OTUB1^fl/fl^ mice, CD11c-Cre OTUB1^fl/fl^ mice produced significantly less IL-12 and TNF upon stimulation with LPS (Fig. 7a), indicating that, in contrast to A20,^12^ OTUB1 in DCs sensitizes mice to LPS by enhancing cytokine production in DCs. Consistently, CD11c-Cre OTUB1^fl/fl^ mice survived significantly longer than OTUB1^fl/fl^ mice (Fig. 7b) due to reduced systemic inflammation.

To explore the role of DC-specific OTUB1 in viral infection, we infected mice with murine cytomegalovirus (MCMV), which induces a strong IL-12 response in various DC subtypes.^67^ In MCMV infection, the induction of IL-12 in DCs is mediated by TLR3,^68^ TLR7,^69^ TLR9,^70,71^ RIG-I-like receptors (RLRs),^72^ and cGAS/STING,^72^ which may compensate for each other with respect to IL-12 production. In contrast to toxoplasmosis and LPS challenge, MCMV infection resulted in comparable DC responses in both the livers (Supplementary Fig. 6a–c) and spleens (Supplementary Fig. 6d–f) of CD11c-Cre OTUB1^fl/fl^ and OTUB1^fl/fl^ control mice. The number of cells in different DC subpopulations (Supplementary Fig. 6b, e) and IL-12-producing DC subsets (Supplementary Fig. 6c, f) was not regulated by DC-specific OTUB1. In addition, serum levels of IL-12, TNF, and IL-6 (Supplementary Fig. 6g) and the viral load in the liver, spleen, lung, and lymph nodes (Supplementary Fig. 6h) were equal in both infected mouse strains.

## Discussion

The swift sensing of invading pathogens by DCs plays a critical role in defense against infections. In this process, TLR-mediated activation of NF-κB is essential for the rapid induction of immune responses. However, hyperactivation of NF-κB signaling may lead to immunopathology and diseases, as observed in LPS-mediated TLR4 activation during Gram-negative sepsis.^73^ The results presented in the present study provide evidence that the DUB OTUB1 critically supports canonical NF-κB activation in DCs and mediates DC-dependent protection in infectious disease, but augments immunopathology upon LPS challenge.

Mechanistically, we identified that UBC13 interacts with OTUB1 upon TgPFN and LPS stimulation, extending the results of a previous study showing that, in the cell nucleus, OTUB1 binds UBC13 in response to DNA double-strand breaks.^42^ With respect to TLR signaling, UBC13 is a key molecule that bolsters MyD88-mediated NF-κB activity by cooperating with the E3 ligases Pellino and TRAF6. First, engagement of TLR11/12 and TLR4 induces recruitment and interaction with MyD88, which is followed by the recruitment of IRAK4 to MyD88. This oligomeric Myddosome acts as a platform for the binding of IRAK1, IRAK2, and IRAK3.^74^ Activated IRAK1 catalyzes the phosphorylation of Pellino, which is a prerequisite for its activity as an E3 ubiquitin ligase.^43,75^ In collaboration with the cognate E2 ubiquitin-conjugating enzyme UBC13, active Pellino acts back against IRAK1 by enabling its K63-specific ubiquitination^43^ and subsequent activation of NEMO. Second, even without K63-linked ubiquitination, IRAK1 can also activate the IKK complex via inducing the association and activation of TRAF6. As an E3 ligase, TRAF6 adds K63-linked polyubiquitin chains to itself, with UBC13 acting as the E2 conjugase.^47^ The bifunctional nature of UBC13-mediated ubiquitination regulation makes UBC13 a pivotal molecule that bolsters MyD88-mediated NF-κB activity. In good agreement with this dual function of UBC13 in MyD88/NF-κB signaling, K63-linked ubiquitination of both IRAK1 and TRAF6 was augmented in TgPFN-stimulated OTUB1-competent BMDCs. Additionally, OTUB1 increased the amount of free ubiquitin in MyD88-activated DCs, which may further contribute to augmented NF-κB activation, since free ubiquitin can directly stimulate TAK1.^53^ The stimulatory effect of OTUB1 on TAK1/NF-κB activation upon TNFR and TLR3 engagement may also be mediated by increased activation of TAK1 by unanchored ubiquitin chains and UBC13-mediated K63-linked polyubiquitination of RIP1, which also contributes to TAK1 activation.^36–38,49^

Interestingly, UBC13 is also a target of ubiquitination, and its protein stability is compromised by the addition of K48-linked polyubiquitin chains by A20.^44^ Here, we identified that K48-linked ubiquitination of UBC13 is counteracted by OTUB1. Since K48-linked ubiquitination is associated with protein degradation, OTUB1 increases the stability of UBC13. In this way, OTUB1 preserves the E2 activity of UBC13 and thereby enhances K63-linked ubiquitination of IRAK1 and TRAF6, leading to increased NF-κB activation. We found that the UBC13 protein is not stable and can be degraded even in the presence of OTUB1 (Fig. 3f, g and Supplementary Fig. 3). However, OTUB1 can significantly delay the degradation of UBC13, showing that OTUB1 serves as a mechanistic agent by which protein ubiquitination and degradation are finely tuned. Interestingly, transfection with OTUB1 mutants revealed that the prevention of UBC13 degradation requires both the N terminus and catalytic domain of OTUB1, given that only wild-type but not the OTUB1-C91S-GFP and the OTUB1-ΔN-GFP mutants rescued UBC13 from degradation (Fig. 3g). Interestingly, consistent with our findings, both the C91 residue and the N terminus of OTUB1 are required for its effect on SMAD3.^20^

Both the canonical NF-κB pathway, which is induced by the engagement of TLRs and dependent on degradation of the NF-κB inhibitor IκBα, and the noncanonical NF-κB pathway, which is dependent on the degradation of the IκB-like inhibitor p100, play fundamental roles in immune responses. Due to the central importance of NF-κB pathways, several DUBs, including TNFAIP3 (A20) and CYLD, critically regulate NF-κB pathways, and each of these DUBs modulates various signaling molecules.^76^ With respect to OTUB1, Li et al.^15^ recently identified that OTUB1 prevented B cell-mediated autoimmunity not only by suppressing p100 degradation and noncanonical NF-κB activation but also by preserving p100 in the steady state, preventing aberrant NF-κB activation in the canonical pathway. In combination with our data demonstrating that OTUB1 supports canonical NF-κB activation in DCs upon stimulation with various proinflammatory stimuli, OTUB1 qualifies as a critical regulator of both canonical and noncanonical NF-κB pathways and their crosstalk depending on the environment, stimulus, and potentially cell type.

In this regard, we identified that OTUB1 played a central protective role in the UBC13-mediated activation of the NF-κB pathway in CD11c^+^ DCs in response to TLR11/12-dependent activation by the protozoal parasite *T. gondii*, but contributed to immunopathology upon TLR4-dependent LPS challenge. Notably, both TgPFN and LPS also induced the stronger activation of MAPKs in OTUB1-competent BMDCs, which is in accordance with the supportive role of UBC13 in LPS-induced MAPK activation.^41^ Thus, these pathways may also contribute to the proinflammatory phenotype of OTUB1^fl/fl^ mice in toxoplasmosis and under LPS challenge, although the NF-κB pathway plays a superior role. Since OTUB1-competent BMDCs showed stronger activation of TAK1 and production of the cytokines IL-12, TNF, and IL-6 upon engagement of TLR2, TLR7, TLR9, and IL-1R, all of which signal via MyD88, and upon TNF stimulation and poly I:C-induced TLR3 activation, both of which induce MyD88-independent signaling, OTUB1 may contribute to proinflammatory DC responses in a wide range of infections and inflammatory disorders. However, our data on OTUB1-independent cytokine production by DCs and pathogen control under MCMV infection illustrate that the underlying pathogen determines the importance of OTUB1 in DC activation. MCMV activates several PRRs in DCs, including TLR3,^68^ TLR7,^69^ TLR9,^70,71^ RLR,^72^ and cGAS/STING,^72^ and these receptors can compensate for each other with respect to their protective DC functions in MCMV infection.^71,72,77^ Additionally, MCMV actively manipulates IRF3 and NF-κB activation to promote viral spread.^78–80^ These data extend those of a previous in vitro study on the inhibition of Sendai virus-induced IFN regulatory factor 3 (IRF3) and NF-κB signaling by OTUB1^28^ and further indicate the interplay between the DC-specific function of OTUB1 with pathogen-mediated modulation of host cell signaling.

To address the importance of OTUB1-mediated NF-κB activation in vivo, we studied a murine model of toxoplasmosis because protection against *T. gondii* is critically dependent on TgPFN-mediated activation of TLR11/12 in DCs. Engagement of TLR11/12 by TgPFN was shown to activate CD8^+^ cDC1 in a MyD88-dependent manner to produce IL-12, which drives protection against *T. gondii* independent of other relevant DC functions, including the cross-presentation of antigens and production of chemokines and other cytokines.^61^ IL-12 production by CD8^+^ cDC1s, the loss of which cannot be compensated for by other IL-12-producing cell populations, is critical for the induction of IFN-γ production by NK cells and antiparasitic effector molecules in infected host cells, resulting in the early containment of parasite multiplication, restriction of the intracerebral parasite load, and increased survival in chronic TE.^61^ Here, we demonstrate that OTUB1 enables DCs to fulfill these critical immunoregulatory functions in toxoplasmosis by enhancing TgPFN/MyD88-induced IL-12 transcription via NF-κB but not IRF8, which also supports IL-12 production by DCs in toxoplasmosis.^5,34^ This assumption was further substantiated by the effects of the inhibition of IL-12 production in both OTUB1-competent and OTUB1-deficient BMDCs upon NF-κB inhibitor treatment. Likewise, OTUB1 promoted LPS-induced TLR4/MyD88-dependent NF-κB activation and cytokine production. Since DCs also play an important role in sepsis and contribute to LPS-induced immunopathology,^12^ mice with DCs with intact OTUB1 function produced significantly more proinflammatory disease-promoting cytokines and succumbed significantly earlier to LPS challenge. It will be of interest to explore in more detail the in vivo role of OTUB1 in other PRR-related signaling pathways, cell types, and infectious diseases.

The balanced activation of DCs in response to invading pathogens is important to ensure pathogen control and to limit immunopathology. DUBs, including A20 and Trabid, have emerged as an important group of enzymes absolutely required for this balanced DC activation.^12–14,81^ In this study, we identified OTUB1 as a novel DUB that regulates TLR-induced DC activation via deubiquitinating and stabilizing UBC13, thereby providing a potential target for the treatment of infectious and inflammatory diseases.

## Materials and methods

### Mice

C57BL/6 OTUB1^fl/fl^ mice^24^ were crossed with C57BL/6 CD11c-Cre mice^32^ to obtain CD11c-Cre OTUB1^fl/fl^ transgenic mice. Genotyping of newborn mice was performed by PCR of tail DNA with primers targeting CD11c-Cre and OTUB1^fl/fl^. Animals were kept under specific pathogen-free conditions in the animal facility at Magdeburg University Hospital (Magdeburg, Germany). All mice were used at 8–12 weeks of age and were sex and age matched. Animal care and experiments were performed according to the European Animal Protection Law and approved by local authorities (Landesverwaltungsamt Halle, Germany; license number 2-1175). The regional Animal Research Ethical Board (Stockholm, Sweden) approved procedures and protocols involving the extraction of cells from mice (N135/15, N78/16), following proceedings described in EU legislation (Council Directive 2010/63/EU).

### *Toxoplasma gondii* infection and in vivo LPS challenge

Mice were infected i.p. with five freshly prepared tissue cysts of the type II DX strain, which was maintained by the chronic infection of NMRI mice and have been prepared from the brain as described before.^82^ Alternatively, mice were infected i.p. with 20,000 or 50,000 GFP-expressing tachyzoites of the type II PTG-GFP strain, which were purchased from ATCC (Manassas, VA, USA) and maintained by in vitro passage in Vero cultures. Upon 60–80% lysis of Vero cell monolayers, cell cultures were mixed by pipetting and subsequently centrifuged at 50 × *g* for 5 min to pellet cellular debris. Thereafter, the supernatant containing tachyzoites was transferred to a new tube and centrifuged at 2000 × *g* for 10 min to spin down the tachyzoites. The number of tachyzoites was determined with a hemocytometer. LPS was purchased from Sigma-Aldrich (Steinheim, Germany), diluted in phosphate-buffered saline (PBS) and i.p. injected at a dose of 10 mg/kg into each mouse.

### Magnetic sorting of leukocytes

Spleens from OTUB1^fl/fl^ and CD11c-Cre OTUB1^fl/fl^ mice were harvested, and a single-cell suspension was obtained by passing the organs through a 70 μm cell strainer. After the lysis of erythrocytes, cells were pelleted by centrifugation at 1200 r.p.m. for 6 min. The cell pellet was resuspended in 40 μl of buffer (PBS supplemented with 0.5% bovine serum albumin (BSA) and 2 mM EDTA, pH 7.2) per 10^7^ cells. DCs, macrophages, NK cells, T cells, and B cells were isolated with specific kits according to the manufacturer’s protocol (STEMCELL Technologies, Cologne, Germany). First, the cells were incubated with fluorescently labeled specific antibody for 15 min. Then, the selection cocktail, which was specific for the fluorochrome used, was added and incubated for 15 min. Finally, the magnetic beads were vigorously mixed and added to the solution for 10 min. The cell suspension was brought to a total volume of 2.5 ml with a medium. Tubes containing the cell suspension were placed inside an EasySep magnet (STEMCELL Technologies) and set aside for 5 min. With one single movement, the magnet-containing tube was inverted, and nonbonded cells were removed from the solution. Cells were then washed with medium, and magnetic isolation was repeated five times. After the last isolation, the tubes were removed from the magnet, and the cells were resuspended in PBS and harvested for further analysis. The purity of the cells was 90–95%, as determined by flow cytometry.

### Cell cultures and transfection

Vero cells were cultured in RPMI medium supplemented with 10% (vol/vol) heat-inactivated fetal calf serum (FCS) and 1% (vol/vol) pen/strep solution in a cell incubator at 37 °C with 5% CO_2_. NIH 3T3 cells were cultured in Dulbecco’s modified Eagle’s medium supplemented with 10% heat-inactivated FCS and 1% pen/strep in an incubator at 37 °C with 5% CO_2_. Control siRNA and OTUB1 siRNA (Thermo Fisher Scientific) were transfected into NIH 3T3 cells with Lipofectamine RNAiMAX transfection reagent (Thermo Fisher Scientific) according to the manufacturer’s instructions. pCMV6-GFP, pCMV6-GFP-OTUB1 (OriGene), pCMV6-GFP-ΔN, and pCMV6-GFP-OTUB1-C91S expression plasmids (generated with a Q5 Site-Directed Mutagenesis Kit, NEB) were transfected into NIH 3T3 cells with Lipofectamine 3000 transfection reagent (Thermo Fisher Scientific) according to the manufacturer’s protocols.

### Bone marrow-derived DCs and bone marrow-derived macrophages

BMDCs were obtained and cultured as described previously.^12^ Cells were differentiated with 35 ng/ml GM-CSF (Peprotech Tebu-bio, Offenbach, Germany) or 400 ng/ml FLT3L (Peprotech Tebu-bio) for 8–10 days. The purity of the BMDC cultures was >90%, as determined by flow cytometry to detect CD11c. To obtain BMDMs, bone marrow was isolated from the femurs of CD11c-Cre OTUB1^fl/fl^ and OTUB1^fl/fl^ mice and then incubated for 8 days with 35 ng/ml M-CSF (Peprotech Tebu-bio). The purity of the BMDM cultures was >90%, as determined by flow cytometry to detect CD11b and F4/80.

### In vitro stimulation

After in vitro expansion, BMDCs were washed once with PBS and plated at the desired concentration with appropriate medium. After 4 h of incubation at 37 °C, the cells were stimulated as indicated. Samples for IP were always stimulated in the presence of the proteasome inhibitor MG132 (Sigma-Aldrich). For the preparation of TLA, freshly released tachyzoites were isolated as described before and then freeze-thawed in liquid nitrogen five times. The TLA concentration was quantified with a spectrophotometer. TgPFN was produced in the Protein Science core facility of the Karolinska Institute (Stockholm, Sweden). The purity of TgPFN was controlled by mass spectrometry, and the protein concentration was determined by Bradford assay. LPS (Sigma, St. Louis, MO, USA) was used at a working concentration of 500 ng/ml. PGN (peptidoglycan), ODN (oligodeoxynucleotide), IMQ (imiquimod), and poly I:C were purchased from Invivogen (San Diego, CA, USA) and used as per the manufacturer’s recommended concentrations. Recombinant murine IL-1β and TNF were purchased from PeproTech (Rocky Hill, USA) and used at working concentrations of 10 and 20 ng/ml, respectively.

### Flow cytometry

Leukocytes were isolated from the peritoneal cavities of mice by lavage with PBS. Intracerebral leukocytes were isolated from brains by a Percoll (GE Healthcare Life Sciences, Marlborough, USA) gradient according to previously published protocols.^83,84^ Cells were counted with a hemocytometer and then stained with fluorochrome-coupled antibodies as indicated. For intracellular staining, cells were stimulated for 6 h with heat-killed (60 °C for 20 min) *T. gondii* (multiplicity of infection (MOI) = 3) in the presence of 1 μg/ml brefeldin A (Thermo Fisher Scientific, Waltham, MA, USA). Anti-CD3 PECy7 (cat. #100220), anti-CD11b PECy7 (cat. #101216), anti-CD8a PECy7 (cat. #100722), anti-CD4 BV421 (cat. #100437), anti-F4/80 BV421 (cat. #123131), anti-PDCA-1 APC (cat. #127016), anti-CD11c APC (cat. #117310), anti-CD45 PerCP (cat. #103130), anti-TNF PE (cat. #506306), anti-mouse IgG PE (cat. #400112), anti-CD11c APCCy7 (cat. #117324), anti-NK1.1 APCCy7 (cat. #108724), anti-CD11b APCCy7 (cat. #101226), anti-B220 BV510 (cat. #103247), anti-CD8 BV510 (cat. #100751), anti-class II MHC PE (cat. #109908), anti-CD80 PE (cat. #104708), anti-CD86 PE (cat. #105008), and anti-CD40 PE (cat. #124610) were purchased from BioLegend (San Diego, CA, USA). Anti-B220 BV421 (cat. #562922), anti-IL-12 APC (cat. #554480), anti-IL-6 PE (cat. #562050), and anti-CD19 APCCy7 (cat. #557655) were purchased from BD Biosciences (San Jose, USA). Anti-IFN-γ APC (cat. #17-7311-82) was purchased from Invitrogen (Carlsbad, CA, USA). The remaining antibodies were purchased from eBioscience (San Diego, USA): anti-CD8 APC (cat. #17-0081-82), anti-Ly6C APC (cat. #17-5932-82), anti-mouse IgG APC (cat. #17-4015-82), anti-NK1.1 PE (cat. #12-5941-82), anti-Ly6G PE (cat. #12-9668-82), anti-CD11c PE (cat. #12-0114-82), anti-IL-12 PE (cat. #12-7123-82), and anti-CD3 APCCy7 (cat. #47-0032-82). Flow cytometry was performed on a FACSCanto II (BD Biosciences), and the data were analyzed with FlowJo^®^ software (FlowJo LLC, Ashland, OR, USA). Cytokines from cell cultures and sera were measured using the BD CBA Mouse Inflammation Kit (BD Bioscience). CBA data were acquired on the FACSCanto II and analyzed with the BD CBA Software (BD Bioscience).

### Quantitative and semiquantitative PCR

DNA and RNA were isolated from mouse tissues or BMDCs. Mouse organs were first mechanically homogenized with the QIAshredder Kit (Qiagen, Hilden, Germany) before the isolation of DNA and RNA using DNeasy and RNeasy Kits (Qiagen), respectively. RNA was then reverse transcribed into complementary DNA with the SuperScript Reverse Transcriptase Kit (Thermo Fisher Scientific). Quantitative and semiquantitative PCR was performed on a LightCycler 480 System (Roche, Ludwigshafen, Germany). The following primers for quantitative PCR of *T. gondii* sequences were custom produced by Eurofins MWG Operon (Ebersberg, Germany): primer sequences 5′→3′; sense GGAACTGATCCGTTCATGAG; antisense TCTTTAAAGCGTTCGTGGTC. The following primers for semiquantitative PCR were purchased from Applied Biosystems (Darmstadt, Germany): OTUB1 (Mm00506597_m1, lot 1189970), UBC13 (Mm00779119_s1, lot 1388107), HPRT (Mm01545399_m1, lot P181210-001), iNOS (Mm00440485_m1, lot 1799604), iGTP (Mm00497611_m1, lot 1467539), GBP3 (Mm00497606_m1, lot 1162514), GBP5 (Mm00463729_m1, lot P160617-002), IL-1β (Mm00434228_m1, lot 1276462), IL-12α (Mm01208555_m1, lot 1139366), IL-12β (Mm99999067_m1, lot P160325-006), IFN-γ (Mm00801778_m1, lot 1272494), TNF (Mn00443258_m1, lot P180926-011), and IL-6 (Mm00446190_m1, lot P140131-000).

### WB analysis

Cells were stimulated as indicated and lysed on ice with complete RIPA lysis buffer (Cell Signaling Technologies, Denver, CO, USA). Nuclear and cytoplasmic fractions were isolated with the Nuclear and Cytoplasmic Extraction Kit (Thermo Fisher Scientific). Protein samples were diluted and heated in Lane Marker Reducing Sample Buffer (Thermo Fisher Scientific) at 95 °C for 5 min. Equal amounts of samples were separated on 8–15% sodium dodecyl sulfate-polyacrylamide gels and subsequently transferred to polyvinylidene difluoride membranes, which were blocked with 5% BSA, followed by incubation with specific antibodies as indicated. Mouse monoclonal anti-HDAC-1 (cat. #Sc-81598), mouse monoclonal anti-UBC13 (cat. #Sc-58452), mouse monoclonal anti-TRAF6 (cat. #Sc-8409), rabbit polyclonal anti-TRAF6 (cat. #Sc-7221), mouse monoclonal anti-Pellino1/2 (cat. #Sc-271065), and mouse monoclonal anti-Uev1a (cat. #sc-390047) antibodies were purchased from Santa Cruz Biotechnology (Dallas, USA). Rabbit polyclonal anti-IRAK1 (cat. #10478-2-AP) and rabbit polyclonal anti-UBC13 (cat. #10243-1-AP) antibodies were purchased from Proteintech Group (Manchester, UK). Rabbit polyclonal anti-OTUB1 antibody (cat. #NBP1-49934) was purchased from Novus Biologicals (Centennial, USA). Rabbit polyclonal anti-phospho-IRAK1 (Thr209) antibody (cat. #SA4505246) was purchased from Sigma-Aldrich. Rabbit polyclonal anti-cIAP1 (cat. #ab2399) was purchased from Abcam (Cambridge, UK). Rabbit monoclonal anti-lysine 48-specific polyubiquitin chains (cat. #05-1307) were purchased from Millipore (Burlington, MA, USA). The following remaining primary antibodies were purchased from Cell Signaling Technologies: rabbit monoclonal anti-GAPDH (cat. #2118), rabbit monoclonal anti-β-actin (cat. #8457), rabbit polyclonal anti-p65 (cat. #3034), mouse monoclonal βTUB (cat. #86298), rabbit monoclonal anti-IFR8 (cat. #5628S), rabbit polyclonal anti-MyD88 (cat. #3699), rabbit polyclonal anti-IRAK4 (cat. #4363), rabbit polyclonal anti-phospho-IRAK4 (cat. #7653S), rabbit polyclonal anti-TAK1 (cat. #4505), rabbit polyclonal anti-phospho-TAK1 (Ser412) (cat. #9339), rabbit monoclonal anti-IκBα (cat. #4812), rabbit monoclonal anti-phospho-IκBα (Ser32) (cat. #2859), rabbit monoclonal anti-phospho-p65 (Ser536) (cat. #4887), rabbit polyclonal anti-A20 (cat. #4625S), mouse monoclonal anti-ubiquitin (cat. #3936), rabbit polyclonal anti-K48 linkage-specific polyubiquitin (cat. #4289), rabbit polyclonal anti-p38 (cat. #9214S), rabbit monoclonal anti-phospho-p38 (Thr180/Tyr182) (cat. #9215S), rabbit polyclonal anti-ERK (cat. #9102S), rabbit polyclonal anti-phospho-ERK (Thr202/Tyr204) (cat. #9101S), rabbit polyclonal anti-SAPK/JNK (cat. #9252), and rabbit monoclonal anti-phospho-SAPK/JNK (Thr183/Tyr185) (cat. #4668). The following secondary antibodies were purchased from Dako (Glostrup, Denmark): swine polyclonal anti-rabbit (cat. #P0217) and rabbit polyclonal anti-mouse (cat. #P0260). WB images were developed with an ECL Plus Kit (GE Healthcare) and captured on an Intas image analysis system (Intas, Göttingen, Germany).

### Immunoprecipitation

Whole-cell lysates of stimulated and unstimulated BMDCs were precleared by incubation with Sepharose G Beads (GE Healthcare) with gentle shaking at 4 °C for 1 h. After removal of the beads by centrifugation, equal amounts of lysates were then incubated with specific antibodies with gentle shaking at 4 °C overnight. The next day, Sepharose G beads were added to the lysates and incubated at 4 °C for 2 h with gentle shaking. To capture the immunocomplex, samples were centrifuged, and beads were washed five times with PBS by pulse centrifugation. The beads were resuspended in 2× Lane Marker Reducing Sample Buffer and boiled at 95 °C for 5 min. Then, the samples were centrifuged at 14,000 × *g* at 4 °C for 1 min, and the supernatants were harvested for WB analysis.

### Free ubiquitin assay

To test unanchored ubiquitin chains in FLT3L-expanded BMDCs, we followed the protocol published by Gilda et al.^57^ One milligram of purified proteins from unstimulated and stimulated samples was depleted of substrate-conjugated ubiquitin chains using TUBEs (UM402, LifeSensors, PA, USA). TUBEs have been shown to be highly efficient at removing polyubiquitinated proteins from lysates.^85^ Two incubation steps with TUBE probes were carried out, and significantly more bait (TUBEs) than required was used to ensure that the lysate had been depleted of ubiquitinated proteins. For every milligram of total protein, 25 μl of resin was utilized and incubated for 1 h at 4 °C with slow movement. TUBE agarose was collected by low speed centrifugation (1000 × *g*, 4 °C) for 2 min. The beads were washed with Tris-buffered saline containing 0.05% Tween-20 and collected by low-speed centrifugation. The supernatant was collected and subsequently analyzed by WB. After pretreatment with 0.5% glutaraldehyde, we used anti-ubiquitin VU-101 antibody (LifeSensors) to detect unanchored ubiquitin chains.

### Transduction of BMDCs

The pLenti-C-Myc-DDK and pLenti-C-Myc-DDK-UBE2N plasmids were purchased from OriGene (Rockville, USA). Lentivirus overexpressing UBC13 and control lentivirus were produced with lentiviral packaging kits (OriGene). At 24 and 48 h after transfection, supernatants containing lentivirus were collected, filtered, aliquoted, and stored at −80 °C. FLT3L-expanded BMDCs were transduced with lentivirus in the presence of 4 μg/ml polybrene (Sigma-Aldrich). The transduction mixture was centrifuged at 1000 × *g* at 32 °C for 90 min and further incubated in the cell incubator for 5 h. Thereafter, the transduced cells were incubated in normal BMDC culture medium for 3 days.

### Motility assays

Motility assays were performed as previously described.^64^ Briefly, DCs were cultured in chamber slides (Lab-Tek^®^, Nalge Nunc International) with complete medium ± freshly egressed GFP-expressing *T. gondii* tachyzoites (type II PTG-GFP, MOI = 3, 4 h incubation). Bovine collagen I (1 mg/ml, Life Technologies) was added, and live-cell imaging was performed for 1 h at 1 frame/min and ×10 magnification (Z1 Observer with Zen 2 Blue v. 4.0.3, Zeiss, Oberkochen, Germany). Time-lapse images were consolidated into stacks, and motility data were obtained from 30 cells/condition (Manual Tracking, ImageJ), yielding mean velocities (Chemotaxis and Migration Tool v. 2.0). GFP^+^ cells harboring tachyzoites were defined as infected cells.

### MCMV infection

Seven- to ten-week-old CD11c-Cre OTUB1^fl/fl^ and OTUB1^fl/fl^ control mice were intravenously infected with 10^6^ MCMV 3DR.^86^ After 36 h, the mice were perfused with PBS; the lymph nodes, lungs, spleens, and livers were removed; and organ homogenates were plated in serial log 10 dilutions on primary murine embryonic fibroblasts. Centrifugal enhancement was performed (2000 r.p.m., 15 min), and after 2 h of incubation at 37 °C and 5% CO_2_, the cells were overlaid with 1% methylcellulose. Plaques were counted after 4 days of culture under a light microscope (Zeiss). In addition, leukocytes were isolated from the spleen and liver and analyzed by flow cytometry. Intracellular staining for IL-12 was performed after 4 h of incubation at 37 °C with GolgiPlug™ (BD Bioscience). Sera were collected from infected animals and used to measure cytokines.

### Histology

At 30 day p.i. with *T. gondii*, anesthetized mice were intracardially perfused with 0.9% NaCl, following which their brains were dissected, snap frozen in isopentane (Flika, Neu-Ulm, Germany), precooled on dry ice, and stored at −80 °C until the preparation of 10-µm-thick frozen sections. Immunohistochemistry was performed with polyclonal rabbit anti-*T. gondii* (BioGenex, Fremont, USA) using an ABC protocol with 3,3′-diaminobenzidine (Merck, Darmstadt, Germany) and H_2_O_2_ as cosubstrates.

### Quantification and statistical analysis

Quantification of WB data was performed using the NIH ImageJ software. Statistical analysis and graphic design were performed using GraphPad Prism 6. Differences for which *P* value <0.05 by Student’s *t* test were considered statistically significant: * indicates a *P* value < 0.05, ** indicates a *P* value < 0.01, and *** indicates a *P* value < 0.001. All experiments were performed at least twice.

## Supplementary information

supplementary figures and legends
