# Supplementary material for: The deubiquitinase OTUB1 augments NF-κB-dependent immune responses in dendritic cells in infection and inflammation by stabilizing UBC13
Source: Cell Mol Immunol. 2020 Feb 5;18(6):1512–27. doi: 10.1038/s41423-020-0362-6 (PMC8167118; doi:10.1038/s41423-020-0362-6)
Supplement: Supplementary file 1 — supplementary figures and legends [file 41423_2020_362_MOESM1_ESM.docx]

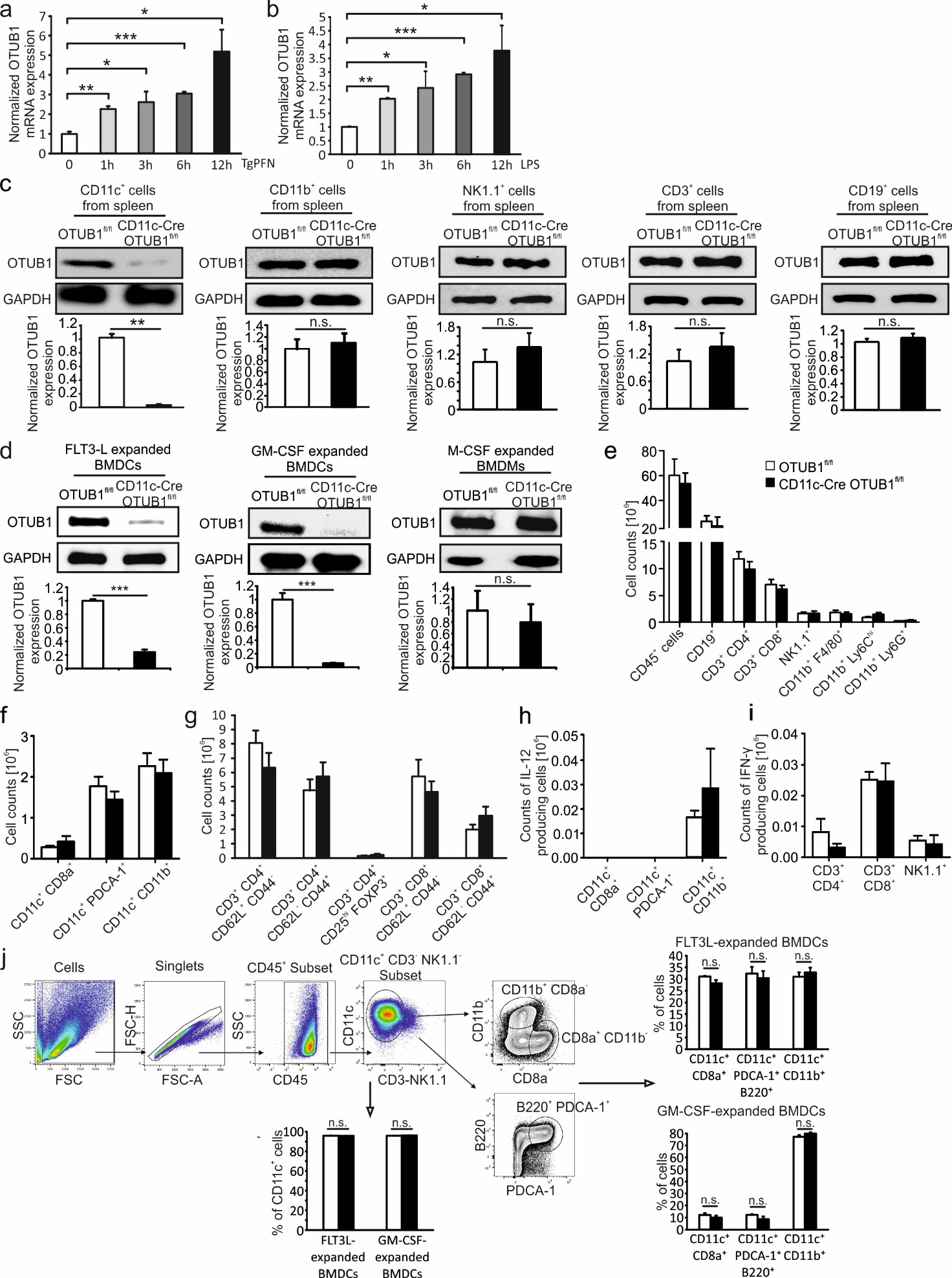


**Supplementary Fig. 1. Characterization of CD11c-Cre OTUB1^fl/fl^ mice in physiological conditions**

(a and b) FLT3L-expanded BMDCs were stimulated with TgPFN (a) and LPS (b) for the indicated time points. OTUB1 mRNA expression was measured by qPCR and normalized to unstimulated sample (n = 3).

(c) CD11c^+^, CD11b^+^, NK1.1^+^, CD3^+^ and CD19^+^ cells were magnetically isolated from spleens of CD11c-Cre OTUB1^fl/fl^ and control OTUB1^fl/fl^ mice, and analyzed by WB for OTUB1 expression. The lower panels show the relative expression of OTUB1 normalized to GAPDH (n = 3).

(d) OTUB1 expression in GM-CSF-expanded BMDCs, FLT3L-expanded BMDCs, and M-CSF-expanded BMDMs was analyzed by WB (upper panels). The lower panels show the relative expression of OTUB1 normalized to GAPDH (n = 3).

(e to g) Absolute numbers of leukocyte (e), DC (f) and T cell (g) subpopulations in the spleen of OTUB1^fl/fl^ and CD11c-Cre OTUB1^fl/fl^ mice were determined by flow cytometry.

(h and i) Absolute numbers of IL-12-producing DCs (h) and IFN-γ-producing T and NK cells (i) were calculated based on flow cytometry results.

(j) Gating strategy for BMDC subpopulations. After *in vitro* FLT3L and GM-CSF expansion, BMDCs were stained for DC markers and measured by flow cytometry. Percentages of total CD11c^+^ cells (lower panel) and CD8a^+^, CD11b^+^ and PDCA-1^+^ B220^+^ subtypes (right panels) were analyzed by FlowJo software (n = 3).

Data are expressed as mean + SD. * p < 0.05, ** p < 0.01, *** p < 0.001.


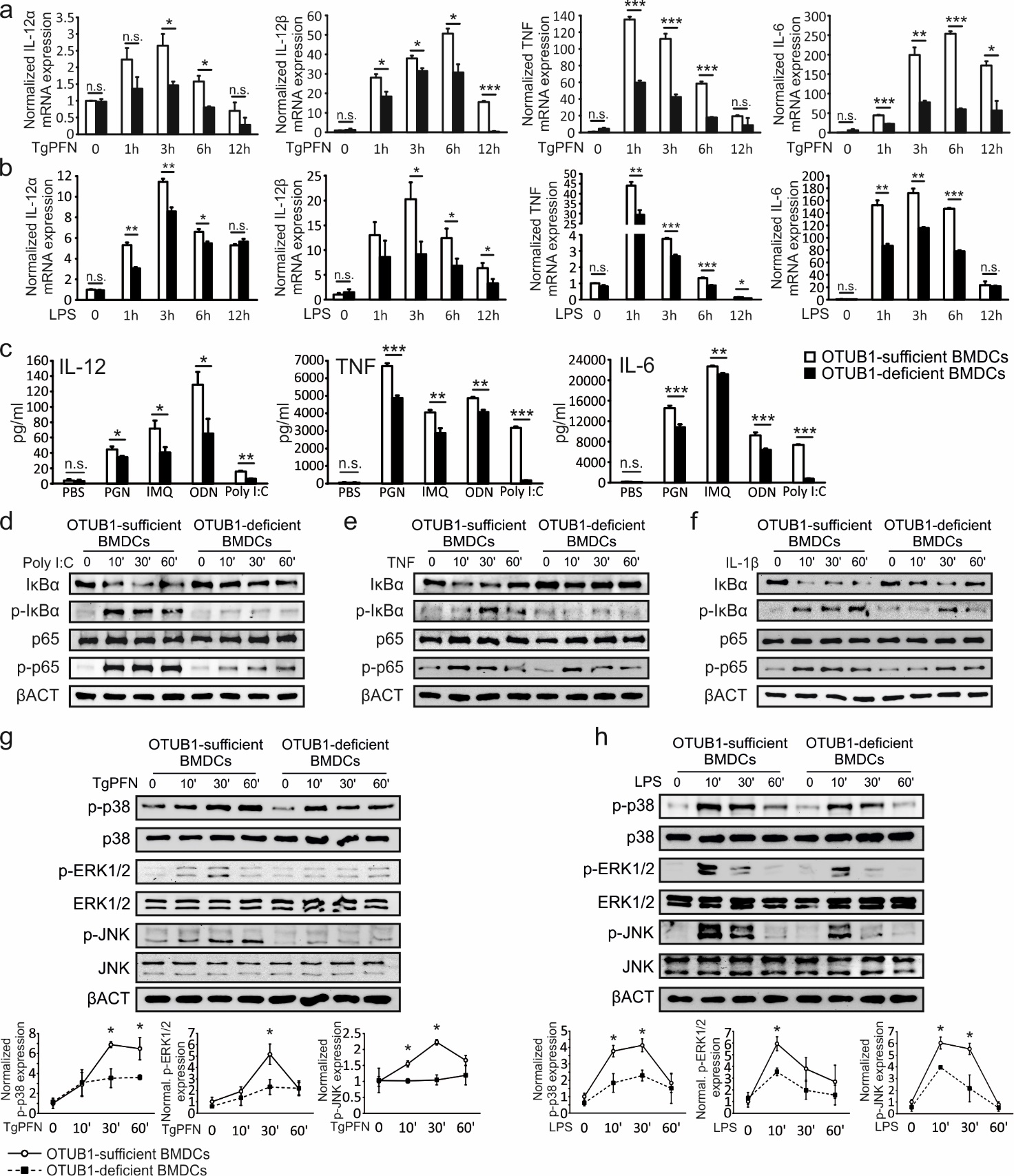


**Supplementary Fig. 2. OTUB1 inhibits TLR2/3/7/9/12-induced cytokine production**

(a and b) FLT3L-expanded BMDC were stimulated with TgPFN (a) or LPS (b) for the indicated time points. mRNA expression of IL-12α, IL-12β, TNF and IL-6 was measured by qPCR. Data show the relative increase over naïve controls.

(c) FLT3L-expanded BMDCs were stimulated with PGN (TLR2 agonist), Imiquimod (IMQ) (TLR7 agonist), CpG ODN (TLR9 agonist) and Poly I:C (TLR3 agonist) for 24 h. Cytokines in the supernatant were analyzed by ELISA (n = 7).

(d to h) FLT3L-expanded BMDCs were stimulated with Poly I:C (d), TNF (e), IL-1β (f), TgPFN (g) or LPS (h) for indicated time points. Whole cell lysates were analyzed by WB with indicated antibodies. Intensity of p-p38, p-ERK1/2 and p-JNK was quantified and normalized to unstimulated samples (n=4).

Data are shown as mean + SD (a-c) or mean + SD (g-h). * p < 0.05, ** p < 0.01, *** p < 0.001.


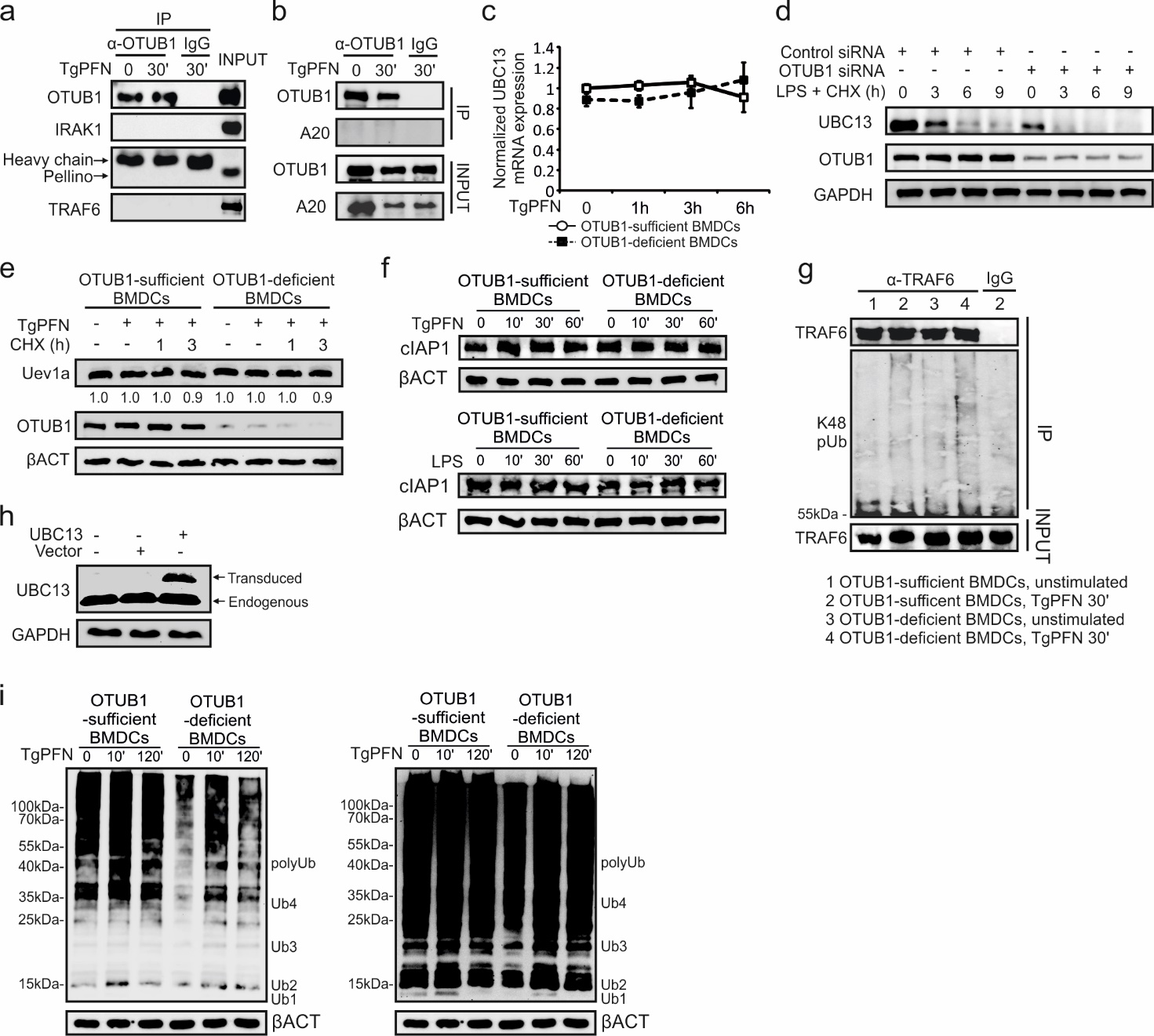
**Supplementary Fig. 3. OTUB1 regulates NF-κB activation by stabilizing UBC13**

(a and b) GM-CSF-expanded BMDCs were left untreated or stimulated with TgPFN for 30 min. Cytoplasmic proteins were isolated and immunoprecipitated with anti-OTUB1 antibodies. Immunoprecipitates and input were analyzed with indicated antibodies.

(c) FLT3L-expanded BMDCs were stimulated with TgPFN for indicated time points. UBC13 mRNA expression was determined by qPCR (n = 4). Data are shown as mean + SD.

(d) NIH 3T3 cells were transfected with siRNA for 36 h. Thereafter, cells were stimulated with LPS and CHX for 0, 3, 6 and 9 h. Protein levels of UBC13 were analyzed by WB.

(e) OTUB1-sufficient and -deficient FLT3L-expanded BMDCs were pre-treated with TgPFN (1 μg/ml) for 1 h or left unstimulated. Then, CHX was added for the indicated time points. Protein levels of Uev1a in whole cell lysates were analyzed by WB.

(f) FLT3L-expanded BMDCs were stimulated with TgPFN (upper panel) or LPS (lower panel) for indicated time points. Whole cell lysates were analyzed by WB with indicated antibodies.

(g) GM-CSF-expanded BMDCs were left unstimulated or stimulated with TgPFN in the presence of MG132. Cytoplasmic proteins were isolated and immunoprecipitated with anti-TRAF6 antibody. Immunoprecipitates and input were analyzed with indicated antibodies.

(h) FLT3L-expanded BMDCs were left untreated or transduced with lentivirus for 72 h. The expression of UBC13 was measured by WB.

(i) GM-CSF-expanded BMDCs were left untreated or stimulated with TgPFN for 30 min. Whole cell lysate were incubated for two times with TUBE-ubiquitin for 1 h and only unbonded proteins were used for WB analysis. Short exposure (left) and long exposure (right) are shown.


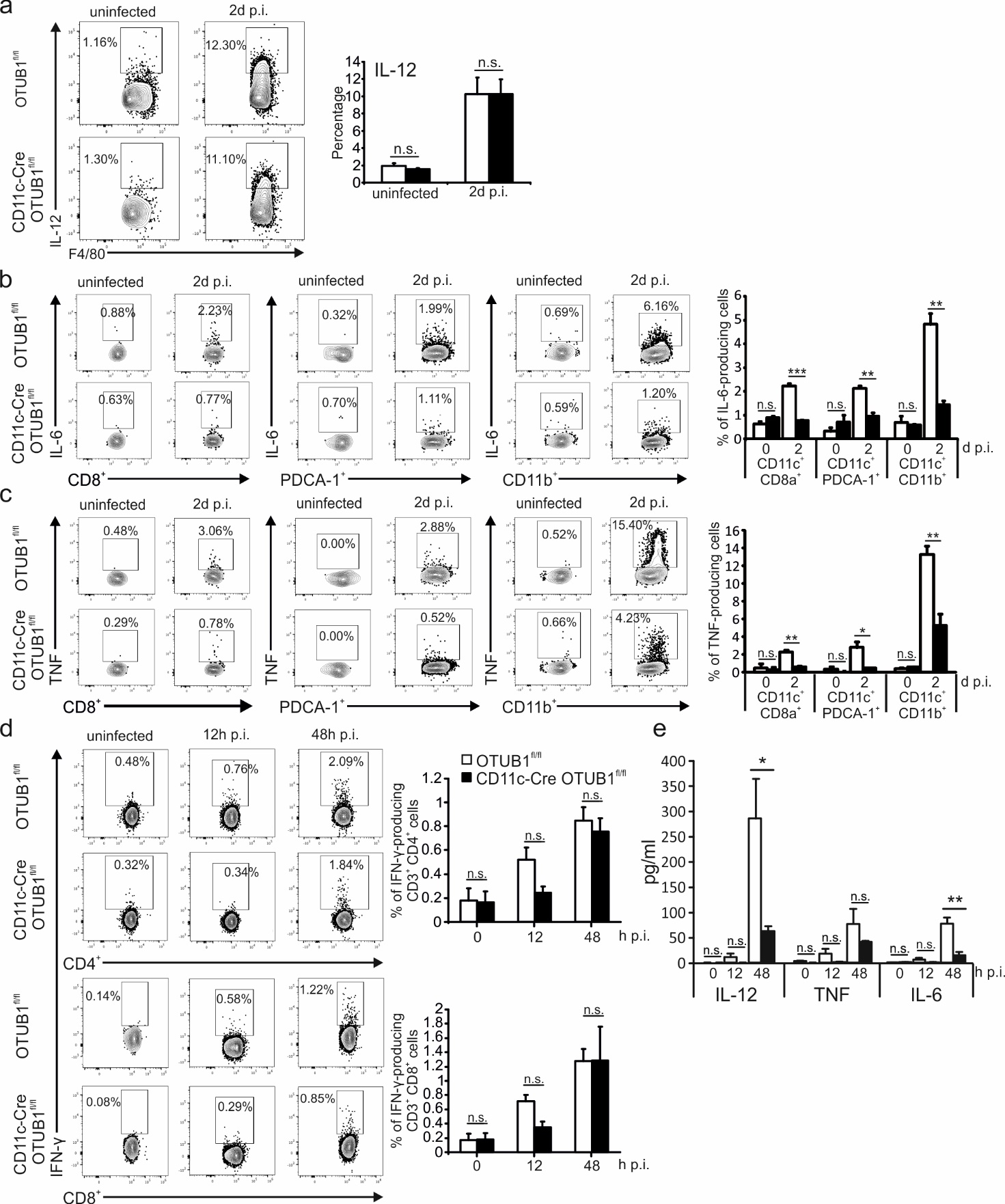
**Supplementary Fig. 4. Cytokine production upon *T. gondii* infection**

(a to c) OTUB1^fl/fl^ and CD11c-Cre OTUB1^fl/fl^ mice were left untreated or infected i.p. with 50.000 tachyzoites for 48 h. Peritoneal cells were collected by lavage of the peritoneal cavity and analyzed by flow cytometry for IL-12-producing macrophages (a), IL-6-producing DCs (b), and TNF-producing DCs (c). Representative flow cytometry plots and statistics are shown (n = 4).

(d and e) OTUB1^fl/fl^ and CD11c-Cre OTUB1^fl/fl^ mice were left untreated or infected i.p. with 50.000 tachyzoites for indicated time points. IFN-γ-producing peritoneal CD4^+^ and CD8^+^ T cells were analyzed by flow cytometry (d). Shown are representative flow cytometry plots and statistics (n = 4). Sera from infected mice were collected and cytokines were measured by ELISA (e) (n = 4).

Data are shown as mean + SD. * p < 0.05, ** p < 0.01, *** p < 0.001.


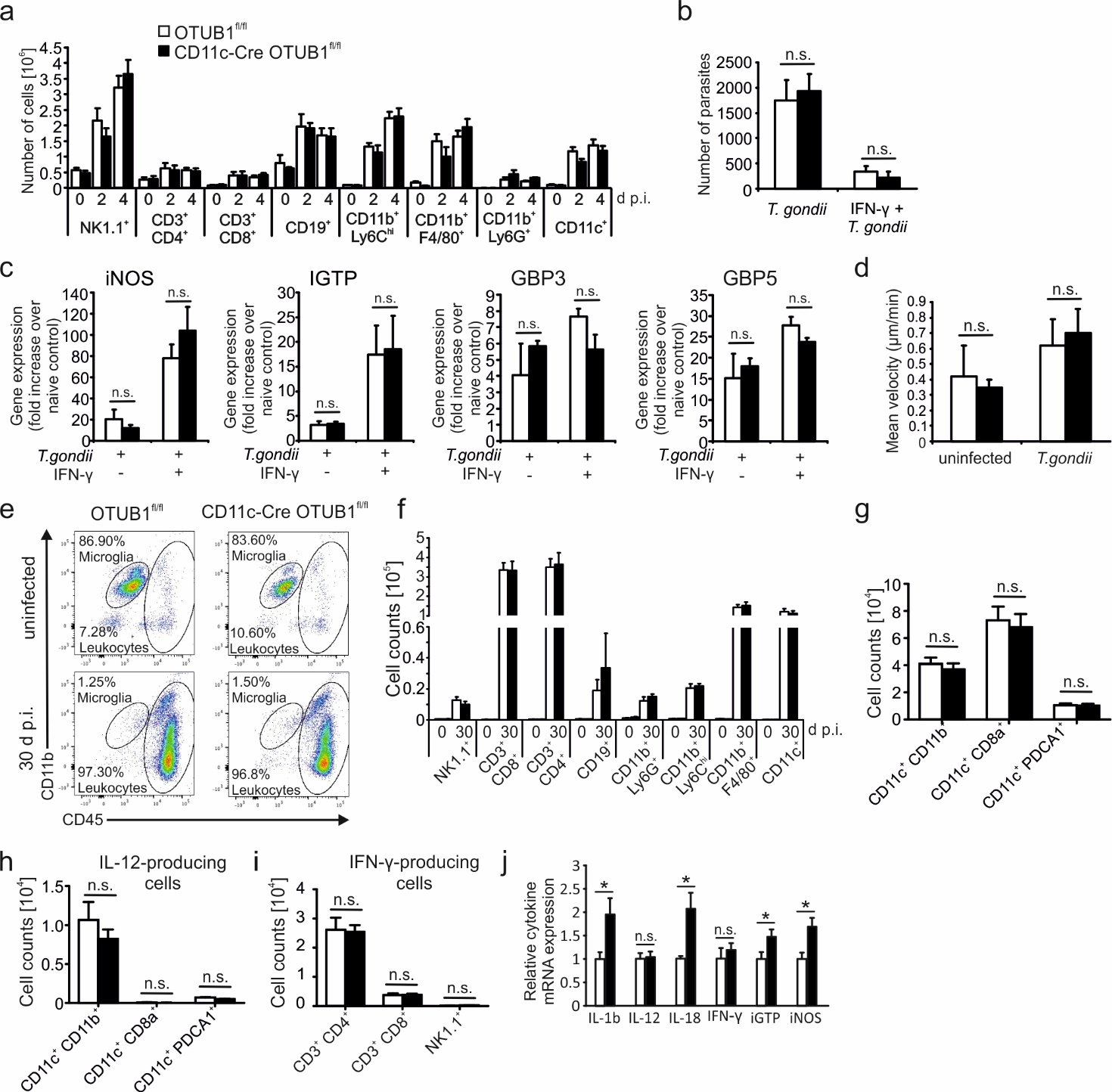
**Supplementary Fig. 5. OTUB1 in DCs is required for surviving toxoplasmosis but is dispensable for leukocyte recruitment, intracellular parasite killing and mobility**

(a) OTUB1^fl/fl^ and CD11c-Cre OTUB1^fl/fl^ mice were infected i.p. with 50.000 tachyzoites for indicated time points. Peritoneal cells were collected by lavage of the peritoneal cavity with PBS and analyzed by flow cytometry (n = 8).

(b and c) OTUB1-sufficient and -deficient BMDCs were untreated or pre-treated with 10 ng/ml of IFN-γ for 24 h followed by infection with *T. gondii* tachyzoites (MOI = 3) for 24 h. Parasite load (b) and anti-parasitic gene expression (c) were determined by PCR (n = 4).

(d) Motility of OTUB1-sufficient and -deficient BMDCs was calculated before and after infection with *T. gondii* tachyzoites at an MOI of 3 (n = 3).

(e to i) OTUB1^fl/fl^ and CD11c-Cre OTUB1^fl/fl^ mice were left untreated or infected i.p. with 3 cysts for 30 days. After that, leukocytes were harvested from the brain and analyzed by flow cytometry for percentages of CD45^+^ cells (e), absolute numbers of leukocyte (f) and DC (g) subsets, as well as absolute numbers of IL-12 (h) and IFN-γ (i) producing cells (n = 4).

(j) Transcription of genes in the brain of OTUB1^fl/fl^ and CD11c-Cre OTUB1^fl/fl^ mice at day 30 p.i. was measured by qPCR (n = 4).

Data are shown as mean + SD. * p < 0.05.


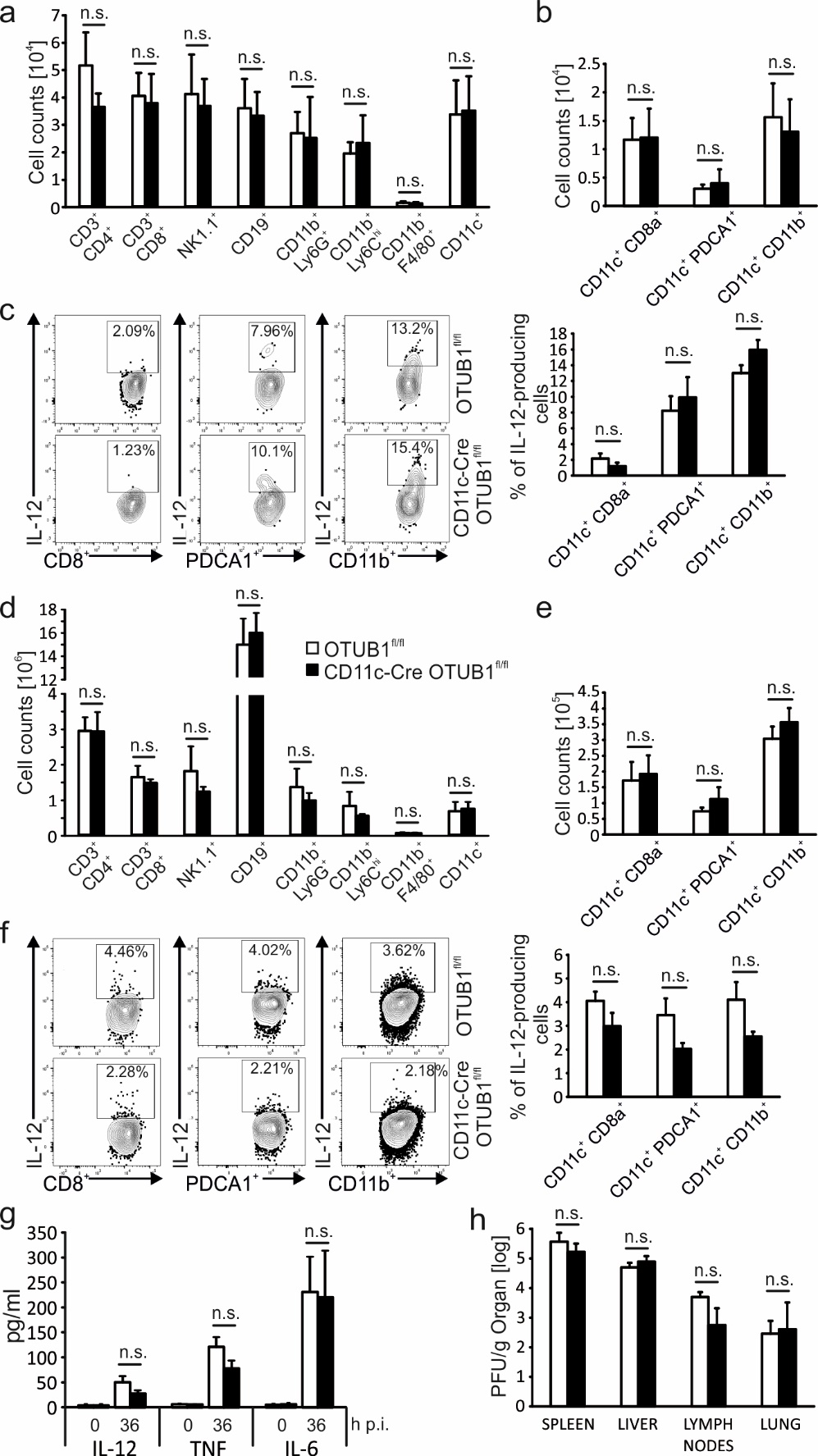


**Supplementary Fig. 6. DC response to MCMV is independent of OTUB1**

(a to h) OTUB1^fl/fl^ and CD11c-Cre OTUB1^fl/fl^ mice were infected i.v. with 10^6^ MCMV and after 36 h mice were sacrificed for the collection of livers, spleens, lungs and lymph nodes (n = 4). (a to c) Flow cytometry analysis of leukocytes isolated from the liver. (d to f) Flow cytometry analysis of leukocytes isolated from the spleen. (g) Cytokines in the serum were measured by ELISA. (h) Organ homogenates were plated in serial log10 dilutions on primary murine embryonic fibroblasts. Plaques were counted after 4 days of culture under a light microscope.

Data are displayed as mean + SD.
